# Arylidenehydrazinyl 4-Methylthiazole-5-carboxylates: Synthesis, Antileishmanial Activity, and Targeting of Trypanothione Synthetase

**DOI:** 10.3390/molecules31132278

**Published:** 2026-06-29

**Authors:** Brunno da S. Souza, Hasnain Mehmood, Estela M. Nolasco, Muhammad Haroon, Tashfeen Akhtar, Nathalia da Silva Brito, Adilson Beatriz, Nikhil Sodhi, Vijay P. Singh, Amilcar Machulek, Gleison A. Casagrande, Dênis Pires de Lima, Sumbal Saba, Thalita B. Riul, Jamal Rafique

**Affiliations:** 1 Laboratório de Síntese Sustentável e Organocalcogênio (LabSO), Instituto de Química (IQ), Universidade Federal de Goiás (UFG), Goiânia 74690-900, GO, Brazil; 2 Department of Chemistry, Mirpur University of Science and Technology (MUST), Mirpur 10250, AJK, Pakistan; 3 Faculdade de Ciências Farmacêuticas, Alimentos e Nutrição (FACFAN), Universidade Federal do Mato Grosso do Sul (UFMS), Campo Grande 79074-460, MS, Brazil; 4 Department of Chemistry, Government Degree College Mirpur, Mirpur 10250, AJK, Pakistan; 5 Instituto de Química (INQUI), Universidade Federal do Mato Grosso do Sul (UFMS), Campo Grande 79074-460, MS, Brazil; 6 Department of Chemistry & Centre of Advanced Studies in Chemistry, Panjab University, Chandigarh 160014, Punjab, India; 7 Instituto de Química, Universidade Federal do Rio Grande do Norte (UFRN), Av. Senador Salgado Filho, s/n, Natal 59078-970, RN, Brazil; machulekjr@gmail.com

**Keywords:** arylidenehydrazinyl thiazole, *Leishmania amazonensis*, molecular docking

## Abstract

Background/Objectives: Leishmaniases are a group of neglected tropical diseases that have been overlooked, and new treatments are needed. This is because the parasites that cause it, from the *Leishmania* genus, have become drug-resistant, and current medications can be toxic. In this regard, arylidenehydrazinyl thiazoles emerge as a potential scaffold for creating new, effective drug candidates to combat this disease. Methods: Here, we report the synthesis of a series of arylidenehydrazinyl thiazole carboxylates (**3a**–**m**, 13 examples, 66–78%) using a simple cost-effective strategy via cyclization of aryl-substituted thiosemicarbazones and ethyl-2-chloro-3-oxobutanoate in an equimolar mixture. These compounds were investigated for their promising bioactive properties. Results: All compounds were fully characterized using spectroscopic techniques (^1^H-, ^13^C-NMR, FTIR spectroscopy, and HRMS) to confirm their purity and identity. These arylidenehydrazinyl thiazole carboxylates have been tested for their cytotoxicity against promastigote and intracellular amastigote forms of *Leishmania amazonensis* (IFLA/BR/1967/PH8) and the NIH/3T3 mouse fibroblast cell lines and their potential interaction with *Leishmania* trypanothione synthetase was explored through in silico molecular docking studies. Conclusions: The compounds showed little or no cytotoxicity against NIH/3T3 fibroblasts, compounds **3a**, **3d** and **3m** showed low cytotoxicity to promastigote forms, but compounds **3b**, **3c**, **3h** and **3m** showed activity against amastigotes (IC_50_ = 15.92 µM for **3m**) at the tested concentrations. In silico molecular docking studies have been deployed to investigate the structural dynamics and the stability of the complex. These findings suggested that the newly developed compounds represent promising preliminary hits for further optimization toward the treatment of diseases associated with *Leishmania* parasites.

## 1. Introduction

Neglected tropical diseases (NTDs) remain a profound and persistent challenge to global health equity, disproportionately affecting the world’s most impoverished and marginalized communities [1,2,3]. Among these NTDs is a collection of conditions known as leishmaniases. These are caused by various species of protozoan parasites from the *Leishmania* genus and are transmitted through the bite of infected sandflies [4,5,6,7].

The burden of leishmaniasis is profoundly concentrated in tropical and subtropical regions, where it disproportionately impacts vulnerable communities with limited access to healthcare and sanitation [8,9,10,11]. The World Health Organization (WHO) estimates that nearly one billion people live in areas endemic for leishmaniasis, with up to one million new cases occurring annually, imposing a catastrophic health, social, and economic burden on approximately two billion people globally [12,13,14,15].

Alarmingly, the epidemiological landscape of leishmaniasis is deteriorating, not improving. Despite ongoing control efforts, the incidence of the disease is rising, with recent studies documenting its spread into regions previously considered non-endemic [16,17,18]. Cutaneous leishmaniasis (CL), the most common clinical form, is at the forefront of this expansion. It is the primary form reported in new cases across vast regions, including the Middle East, North Africa, Asia, and Europe, and remains highly prevalent throughout Latin America [19,20,21,22].

The burden of CL is twofold, encompassing both a deep social and a critical therapeutic crisis. Socially, the disease is highly stigmatizing; its often disfiguring skin lesions can lead to severe psychological distress, social exclusion, and economic hardship, as scars are visibly associated with poverty and illness [23,24,25,26]. This stigma is intensified by the disease’s strong link to social vulnerability, where predisposing factors like malnutrition, poor housing, and limited access to healthcare create a vicious cycle of infection and marginalization [27,28,29,30].

Therapeutically, this crisis is compounded by the severe limitations of existing pharmacologic arsenals [31,32,33,34]. First-line treatments, primarily pentavalent antimonials, are plagued by the necessities of prolonged, parenteral administration and a high frequency of severe adverse effects, including cardiotoxicity and nephrotoxicity, which often outweigh their clinical benefits [35,36,37]. Second-line options, such as amphotericin B, pentamidine, and miltefosine, offer no panacea, being limited by high cost, variable efficacy, emerging parasite resistance, and significant toxicity that precludes their safe use in resource-poor settings [38,39,40].

Heterocyclic scaffold holds an important place in modern pharmaceutical industries, as more than 70% of major commercially available synthetic drugs contain at least one heterocyclic scaffold [41,42,43]. In addition, *N*-heterocyclic compounds occupy a prominent place in medicinal chemistry since more than half of the FDA-approved small-molecule therapeutics contain at least one of these structures at their core [44,45]. In this regard, thiazole nucleus has gained importance because of their structural diversity.

**Figure 1:**
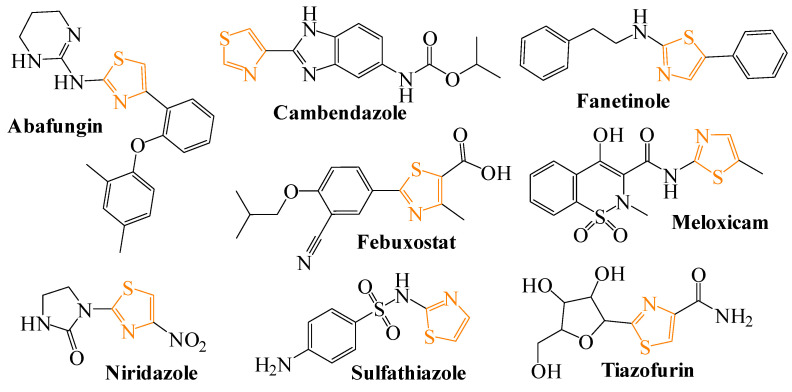
Commercially available drugs with thiazole nucleus.

Among thiazoles structures, 1,3-thiazole is a five-membered compound that contains both sulfur and nitrogen in a heterocyclic ring [46,47]. It constitutes a core skeleton of various commercially available drug candidates and the main unit in a diverse range of important scaffolds that are central to several fields of science, biological and medicinal systems [48]. The chemistry of thiazoles was widely developed with respect to anti-microbial, anti-cancer, anti-tubercular, antioxidant, anti-inflammatory, and other therapeutically active agents [49,50,51,52,53,54,55,56,57,58]. Figure 1 shows selected examples of commercially available drugs and bioactive molecules based on thiazole nucleus [46,48,59,60].

A common strategy for designing new compounds with anti-Trypanosomatidae activity is to target enzymes that are essential to the parasite’s metabolism. One such enzyme is trypanothione synthetase (TryS), which plays a key role in the synthesis of antioxidant molecules. Together with trypanothione reductase (TryR), TryS is crucial for maintaining redox balance and, consequently, for the survival of Trypanosomatidae parasites [61,62]. In this regard, compounds containing a thiazole ring with various modifications have been studied in silico and in vitro against *Leishmania* and *Trypanosoma* parasites.

As part of our research interest in synthesis and biological evaluation of heterocyclic compounds [63,64,65,66,67,68,69,70] and with the aim of advancing drug discovery of new antileishmanial agents [71,72], here, we describe a new method for the preparation of arylidenehydrazinyl 4-methylthiazole-5-carboxylates derivatives. Moreover, their cytotoxicity against promastigote and intracellular amastigote forms of *Leishmania amazonensis* and the NIH/3T3 mouse fibroblast cell lines has been described. Furthermore, in silico molecular docking studies were also explored as a preliminary computational approach to investigate the interaction patterns of selected compounds with target proteins of *Leishmania* trypanothione synthetase.

## 2. Results and Discussion

### 2.1. Chemistry

**Scheme 1:**
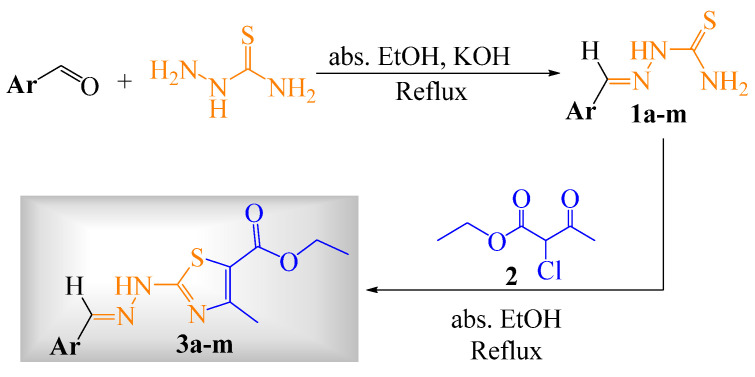
Synthetic route for the arylidenehydrazinyl 4-methylthiazole-5-carboxylates derivatives.

The targeted compounds (**3a**–**m**) were synthesized through the method shown in Scheme 1, with respective structures and yields in Table 1. The procedure involves refluxing equimolar mixture of aryl-substituted thiosemicarbazones (**1a**–**m**) and ethyl 2-chloro-3-oxobutanoate (**2**) in absolute ethanol. A colored heterocyclic product formed upon pouring in crushed ice. 

The formation of cyclized product was first indicated by color changes, melting point and chromatography R_f_ values (provided in experimental data for each compound). Functional group analysis was carried out through IR spectroscopy. Structural exposition of each individual compound was attained through ^1^H and ^13^C NMR spectroscopy. The spectroscopic data is consistent with proposed structure, confirmed by HRMS.

IR spectra indicated the key connections (functional groups) in the synthesized compounds. The presence of ester group, azomethine carbon and methyl group bonded at the fourth position of the thiazole ring is indicated by the absorption band in the range of 1699–1605 cm^−1^, 1599–1549 cm^−1^ and 2984–2901 cm^−1^ respectively. The same linages were supported by ^1^HNMR spectroscopy. A triplet of three protons in the range of 1.27–1.39 ppm and a downfield quartet of two protons in the range of 4.20–4.33 ppm are an indication of the ethyl group of the ester. A singlet of three protons in the range of 2.22–2.68 ppm is ascribed to the methyl group at C4 of thiazole. A characteristic signal of the azomethine proton was observed in the range of 7.71–8.41 ppm in all cases. The proposed structures were further verified through ^13^CNMR spectroscopy. Characteristic signals in the ^13^CNMR spectrum include C2, C4, C5 of thiazole, carbonyl carbon and azomethine carbon. These carbons were observed in the range of 167.4–170.9 ppm, 149.9–159.1 ppm, 110.2–128.9 ppm, 161.9–162.9 ppm and 132.8–153.7 ppm respectively. The calculated masses of the proposed structures are in good agreement with observed masses in the HRMS.

### 2.2. Biological Assays

The compounds were tested for cytotoxicity against promastigote and amastigote forms of *Leishmania* (L.) *amazonensis* (IFLA/BR/1967/PH8) and the NIH/3T3 mouse fibroblast cell lines. All compounds showed little or no cytotoxicity against NIH/3T3 fibroblasts at the concentrations tested (15.625 to 250 µM), as shown in Table 2. Only compounds **3d**, **3e** and **3m** reduced cell viability at higher concentrations, and CC_50_ values were obtained (Figure S55). These results indicate that, in general, the compounds were not toxic to the cell line tested, a promising factor for in vitro biological activity studies.

As for the promastigote forms of *L. amazonensis*, some compounds reduced parasite´s viability at the higher concentrations tested (1.56 to 25 µM), Table 2, as noted for compounds **3d** and **3m** (Figure S56). Considering the small reduction in viability observed, the IC_50_ values were obtained only for compound **3a** (Table 2). Two reference drugs for the leishmaniasis treatment, amphotericin B and pentamidine, were also evaluated as controls in this experiment, and their IC_50_ are also in Table 2 and Figure S58. It is important to note that compounds displaying little or no in vitro activity against promastigote forms of *L. amazonensis* may still exhibit significant activity against intracellular amastigotes, which represent the clinically relevant form found in vertebrate hosts [73].

To this end, an experiment was conducted in which murine macrophages infected with *L. amazonensis* were treated with compounds **3a**–**m** to evaluate their effect on intracellular amastigotes. This experimental model was designed to mimic in vivo infection environment, in which the compounds must penetrate infected macrophages and reduce the intracellular parasite burden. Four of the compounds exhibited activity against intracellular amastigotes: **3m** was the most active, followed by **3c**, **3h** and **3b** (see Table 2 and Figure S57).

A preliminary structure–activity relationship (SAR) analysis was conducted based on the antiamastigote results obtained for the series **3a**–**m**. The common scaffold—an arylidenehydrazinyl 4-methylthiazole-5-carboxylate—was held constant across all derivatives, and biological differences were thus attributed to the nature and substitution pattern of the arylidene moiety. Among the inactive or poorly active compounds, those bearing electron-donating methyl groups (**3f**), methoxy groups in symmetric or meta positions (**3i**, **3j**), a hydroxyl/methyl combination (**3e**), or a bithiazole moiety (**3l**), showed no meaningful activity against amastigotes, suggesting that these substituents do not favor intracellular parasite inhibition. In contrast, the four active compounds revealed interesting trends: **3b** and **3c**, bearing 2,6-dichlorophenyl and 3,4-dichlorophenyl substituents, respectively, displayed comparable antiamastigote IC_50_ values (~32–34 µM), indicating that the presence of two chlorine atoms on the aryl ring is favorable, regardless of their relative position. Compound **3h**, featuring a 2,4-dimethoxyphenyl group, also exhibited similar activity (~33.6 µM), suggesting that electron-donating ortho/para methoxy groups may also confer antiamastigote activity, possibly through enhanced membrane permeability. Most notably, compound 3m, bearing a 3-bromothiophen-2-yl heterocyclic substituent instead of a phenyl ring, was the most potent derivative of the series (IC_50_ = 15.92 µM), highlighting the advantage of replacing the benzene ring with a bromine-substituted thiophene. The combination of the electron-withdrawing bromine atom and the sulfur-containing heteroaromatic system appears to positively modulate both lipophilicity and the capacity to interact with intracellular targets, consistent with its superior docking score against trypanothione synthetase (binding energy: −5.37 kcal/mol, Table 3). Overall, these findings suggest that halogen substitution—particularly on heteroaromatic rings—and ortho/para-directed electron-withdrawing groups are important structural features for antileishmanial activity in this series and should be prioritized in future structural optimization efforts.

Compound **3m** showed low activity against promastigotes but was the most active against intracellular amastigotes of *L. amazonensis*, with the lowest IC_50_ obtained for this series. It also exhibited good selectivity, as shown in Table 2 by the selectivity index (SI) of 7.6, calculated as the ratio of CC_50_ to IC_50_. Although macrophage cytotoxicity would be the most biologically matched comparator for the intracellular amastigote assay, SI values are commonly calculated against different mammalian cell types in antileishmanial screening. We therefore report the NIH/3T3-based SI as a general measure of mammalian selectivity, while acknowledging that a macrophage CC50 would provide a more assay-matched denominator [74]. Compounds **3c**, **3h** and **3b** showed similar antiamastigote activity, whose IC_50_ values ranged from 32.86 to 34.18 µM. Nevertheless, the results obtained in the present study, while encouraging, remain modest: compound **3m**, the most active derivative of the series, exhibited antiamastigote activity with an IC_50_ of approximately 15 μM, inferior to reference drugs such as amphotericin B and pentamidine, also used as controls here, with IC_50_ also in Table 2 results are similar to those in the recent literature regarding compounds containing thiazoles moieties. One study evaluated the synthesis and antiparasitic activity of 13 new thiosemicarbazones and 16 naphthyl-thiazole derivatives [75]. Against *L. amazonensis*, the thiazoles exhibited IC_50_ values between 16.48 and 247.59 µM for promastigote forms. In assays involving amastigote forms of *Leishmania*, IC_50_ values ranged results were recorded from 50.80 to 379.25 µM for thiazoles [5].

Another investigation explored the biological potential of eight 4-(4-chlorophenyl)thiazole compounds containing a naphthyl moiety [76]. For *L. amazonensis*, these molecules exhibited IC_50_ values ranging from 19.86 to 200 µM for promastigotes. However, activity against *Leishmania* amastigotes was characterized as moderate to inactive, with IC_50_ values between 101 and over 200 µM [76], well above the values obtained for our compounds.

In *Leishmania* parasites, trypanothione plays a critical role in defending against oxidative stress by neutralizing reactive oxygen species (ROS) produced by both the parasite and the host [77]. As trypanothione and its related enzymes (like trypanothione synthetase and reductase) are absent in humans, they present promising targets for antileishmanial therapies [78]. Disrupting trypanothione synthesis or function weakens the parasite’s antioxidant defenses, leading to its death without affecting human cells [79].

Targeting the trypanothione system has emerged as an effective strategy for the development of selective anti-*Leishmania* agents with reduced side effects. In the present study, a series of the most active compounds **3b**, **3c**, **3h**, **3i**, **3k**, **3m** and pentamidine was evaluated through molecular docking to preliminarily assess their binding interactions with *Leishmania* trypanothione synthetase (PDB ID: 2VOB) using AutoDock 4.2 [80]. The protein structure was prepared by removing water molecules and adding polar hydrogens and Kollman charges. The grid box was centered on the active site defined by the co-crystallized ligand to ensure inclusion of key catalytic residues. Docking simulations were carried out using the Lamarckian Genetic Algorithm (LGA) implemented in AutoDock 4.2 with multiple runs to ensure reproducibility and convergence of results.

The in silico docking results demonstrated that the binding energies of the tested compounds ranged from −3.16 to −5.37 kcal/mol compared to the reference compound pentamidine (−2.96 kcal/mol). These results suggest favorable predicted binding affinities and provide computational support for further investigation these compounds as potential anti-*Leishmania* agent.

**Figure 2:**
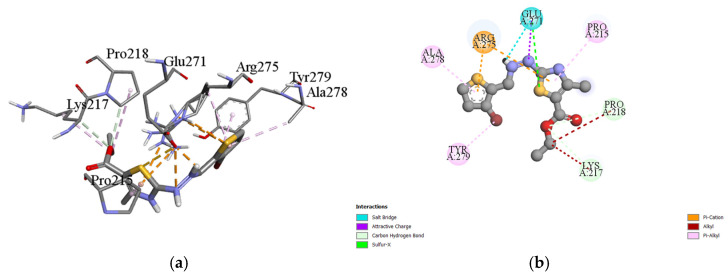
(**a**) 3D and, (**b**) 2D binding model of compounds **3m**.

The molecular interactions between compounds **3b**, **3c**, **3h**, **3i**, **3k**, **3m** and the 2VOB protein from *Leishmania* were examined as shown in Figure 2 (see Figures S53 and S54 in the Supporting Information). These interactions involved hydrogen bonds, π-alkyl, π-sigma, amide-π interactions, and van der Waals forces. These interactions included hydrogen bonding, π-alkyl, π-sigma, amide-π stacking, and van der Waals forces. Table 3 summarizes the binding energies, inhibition constants, and the key amino acid residues involved in hydrogen bond formation. Importantly, the docking results against trypanothione synthetase indicate a possible interaction; however, these findings are considered supportive and hypothesis-generating with respect to the observed antileishmanial activity and do not establish a direct mechanistic link. Direct biochemical evidence of enzyme inhibition, such as enzymatic assays or target engagement studies, would be required to confirm trypanothione synthetase as the molecular target of these compounds.

It is important to emphasize that while molecular docking provides valuable insights into potential binding modes and interactions, these computational predictions require experimental validation. The binding energies and interaction patterns reported herein should be considered as hypothesis-generating data that support further investigation of trypanothione synthetase as a potential target, rather than as definitive evidence of enzyme inhibition.

Interestingly, the compounds with the lowest binding energies for 2VOB protein showed no cytotoxicity for NIH/3T3 fibroblasts or promastigote forms of *L. amazonensis*. However, two compounds, **3b** and **3h**, exhibited activity against intracellular amastigote forms of *L. amazonensis*, as shown in Table 2. While the binding energies listed in Table 3 suggest possible interactions with trypanothione synthetase, this remains a hypothesis that requires experimental validation through enzymatic assays. Furthermore, the molecular properties and aryl substituents of these compounds may facilitate their entry into macrophages and contribute to the observed antileishmanial activity through mechanisms that may or may not involve TryS inhibition.

The distinct metabolic, biochemical, and biological variations between promastigote and amastigote forms throughout *Leishmania*’s lifecycle [81] may account for differences in enzymatic inhibitory and antileishmanial activities observed across cell types [82]. In this context, the use of intracellular amastigotes provided essential information on the capacity of these compounds to target the clinically relevant stage of the parasite [83], and the partial corroboration of this activity through in vitro interaction with trypanothione synthetase (2VOB) supports a plausible mechanism of action, highlighting this enzyme as a potential target for this chemical class. Taken together, these findings suggest that compound **3m** should be regarded as a preliminary hit, and that structural optimization guided by the present pharmacological and enzymatic data may lead to new synthetic molecules with improved potency and selectivity against *Leishmania*.

## 3. Materials and Methods

### 3.1. Synthesis of Ethyl-(E)-2-(2-arylidenehydrazineyl)-4-methylthiazole-5-carboxylates

#### 3.1.1. Materials

All the reagents and solvents used for the synthesis of arylidenehydrazinyl thiazole carboxylates (**3a**–**m**) were of analytical grade and were used without any further purification. The reagents and solvents were purchased from reputed chemical suppliers, i.e., Sigma Aldrich, Riedel-de-Haen, and Analar. Reaction monitoring and retention factor (*Rf*) determination were performed on pre-coated thin layer chromatography (TLC) sheets (Silica gel 60 F_254_, Merck, Darmstadt, German), which were visualized either through quenching of ultraviolet or fluorescence light (λ_max_ = 254 and 366 nm, respectively). A digital melting point apparatus was used to determine the uncorrected melting points of all solid compounds. Final products (**3a**–**m**) were characterized using the ^1^H and ^13^C NMR spectra on 400 MHz (^1^H: 400 MHz; ^13^C: 100 MHz) and 500 MHz (^1^H: 500 MHz; ^13^C: 125 MHz) spectrometers, having the residual solvent peaks of CDCl_3_ (^1^H: *δ* 7.26; ^13^C: *δ* 77.2), DMSO-*d*_6_ (^1^H: *δ* 2.50; ^13^C: *δ* 39.7), as an indirect reference to tetramethyl silane (TMS). The electron ionization (EI) technique was utilized to set up the time of flight (TOF) equipment used to generate the high-resolution mass spectra (HRMS) operating in the positive ion mode.

All reactions were performed in a well-ventilated fume hood under an inert environment using a Schlenk line. The starting materials, aryl-substituted thiosemicarbazones **1a**–**m**, were prepared according to our previous report [84].

#### 3.1.2. General Procedure for the Synthesis of Arylidenehydrazinyl Thiazole Carboxylates (**3a**–**m**)

The arylidenehydrazinyl thiazole carboxylate derivatives were prepared by refluxing together aryl-substituted thiosemicarbazones **1a**–**m** (1 mmol) with equimolar amount of ethyl 2-chloro-3-oxobutanoate **2** in absolute ethanol (15 mL) for a duration of 5 h. Reaction progress was monitored through TLC and upon completion; the reaction was quenched with ice cold water, resulting precipitation. The resulting precipitates were filtered and successively washed with copious deionized water to afford final product **3**, which were dried under vacuum. Lastly, purity was rechecked via TLC.

#### 3.1.3. Spectral Data of Synthesized Products (**3a**–**m**)

***Ethyl (E)-4-methyl-2-(2-((5-methylfuran-2-yl)methylene)hydrazineyl)thiazole-5-carboxylate* (3a)**. Off-white solid; yield: 72%; melting point: 187–189 °C; R_f_: 0.55 (acetone/*n*-hexane, 1:3); FTIR (ATR) cm^−1^: 3185 (N-H stretching), 3066 (=C-H stretching), 2922 (C-H aliphatic stretching), 1683 (C=O stretching), 1582 (C=N stretching), 1539, 1522, 1366, 1313 (C=C aromatic ring stretching), 1424 (C-H aliphatic bending), 1088 (C-O stretching), 1012-734 (characteristic of thiazole); ^1^H-NMR (400 MHz, CDCl_3_): *δ* (ppm) 7.71 (s, 1H, H-C=N-), 6.59 (d, 1H, furan-H, *J* = 4.6 Hz), 6.10 (d, 1H, furan-H, *J* = 3.4 Hz), 4.30 (q, 2H, -CH_2_-CH_3_, *J* = 7.1 Hz), 2.60 (s, 3H, -CH_3_), 2.40 (s, 1H, -CH_3_), 1.37 (t, 3H, CH_3_-CH_2_, *J* = 7.1 Hz); ^13^C-NMR (100 MHz, CDCl_3_): *δ* (ppm) 170.0 (Thiazole C2), 162.6 (C=O), 156.7 (thiazole C4), 155.4 (furan C5), 147.5 (furan C2) 134.6 (H-C=N- azomethine), 114.6 (thiazole C5), 111.0 (furan C3), 108.4 (furan C4), 60.6 (CH_2_-O-), 17.1 (CH_3_-), 14.4 (CH_3_-CH_2_), 13.9 (-CH_3_); HRMS: *m*/*z* calculated for C_13_H_15_N_3_O_3_S [M+H]^+^: calculated 294.0907, found 294.0909, [M + Na]^+^: calculated 316.0726, found 316.0723, [2M + Na]^+^: calculated 609.1560, found 609.1551.

***Ethyl (E)-2-(2-(2,6-dichlorobenzylidene)hydrazineyl)-4-methylthiazole-5-carboxylate* (3b)**. Off-white solid; yield: 75%; melting point: 201–202 °C; R_f_: 0.64 (acetone/*n*-hexane, 1:3); FTIR (ATR) cm^−1^: 3136 (N-H stretching), 3988 (=C-H stretching), 2901 (C-H aliphatic stretching), 1699 (C=O stretching), 1580 (C=N stretching), 1549, 1430, 1370, 1313 (C=C aromatic ring stretching), 1409 (C-H aliphatic bending), 1086 (C-O stretching), 975-755 (characteristic of thiazole); ^1^H-NMR (400 MHz, CDCl_3_): *δ* (ppm) 8.27 (s, 1H, H-C=N), 7.39 (d, 2H, Ar-H, *J* = 8.0 Hz), 7.22 (d, 1H, Ar-H, *J* = 8.0 Hz), 4.31 (q, 2H, -CH_2_-CH_3_, *J* = 7.2 Hz), 2.67 (s, 3H, -CH_3_), 1.37 (t, 3H, CH_3_-CH_2_, *J* = 7.2 Hz); ^13^C-NMR (100 MHz, CDCl_3_): *δ* (ppm) 170.9 (thiazole C2), 162.6 (C=O), 156.7 (thiazole C4), 139.3 (C=N, azomethine), 135.0, 130.0, 129.4, 129.2 (Ar-C), 111.8 (thiazole C5), 60.8 (CH_2_-O), 17.3 (-CH_3_), 14.4 (CH_3_-CH_2_); HRMS: *m*/*z* calculated for C_14_H_13_Cl_2_N_3_O_2_S [M + H]^+^: calculated 358.0178, found 358.0168, [M + Na]^+^: calculated 379.9998, found 379.9994, [2M + Na]^+^: calculated 737.0103, found 737.0077.

***Ethyl (E)-2-(2-(3,4-dichlorobenzylidene)hydrazineyl)-4-methylthiazole-5-carboxylate* (3c)**. Light yellow solid; yield: 73%; melting point: 214–216 °C; R_f_: 0.60 (acetone/*n*-hexane, 1:3); FTIR (ATR) cm^−1^: 3200 (N-H stretching), 3093 (=C-H stretching), 2934 (C-H aliphatic stretching), 1656 (C=O stretching), 1562 (C=N stretching), 1473, 1368, 1329 (C=C aromatic ring stretching), 1432 (C-H aliphatic bending), 1096 (C-O stretching), 1029-728 (characteristic of thiazole); ^1^H-NMR (400 MHz, CDCl_3_): *δ* (ppm) 7.81 (s, 1H, H-C=N), 7.73 (d, 1H, Ar-H, *J* = 1.9 Hz), 7.49–7.42 (m, 2H, Ar-H), 4.20 (q, 2H, -CH_2_-CH_3_, *J* = 7.2 Hz), 2.57 (s, 3H, -CH_3_), 1.34 (t, 3H, CH_3_-CH_2_, *J* = 7.2 Hz); ^13^C-NMR (100 MHz, CDCl_3_): *δ* (ppm) 170.0 (thiazole C2), 162.0 (C=O), 155.9 (thiazole C4), 141.7 (C=N, azomethine), 134.0, 133.6, 130.8, 128.2, 126.1 (Ar-C), 111.9 (thiazole C5), 60.9 (CH_2_-O), 16.7 (CH_3_-), 14.4 (CH_3_-CH_2_); HRMS: *m*/*z* calculated for C_14_H_13_Cl_2_N_3_O_2_S [M + H]^+^: calculated 358.0178, found 358.0178, [M + Na]^+^: calculated 379.9998, found 379.9995, [2M + Na]^+^: calculated 737.0103, found 737.0087.

***Ethyl (E)-2-(2-(2,4-bis(trifluoromethyl)benzylidene)hydrazineyl)-4-methylthiazole-5-carboxylate* (3d)**. Off-white solid; yield: 71%; melting point: 212–214 °C; R_f_: 0.52 (acetone/*n*-hexane, 1:3); FTIR (ATR) cm^−1^: 3206 (N-H stretching), 3101 (=C-H stretching), 2984 (C-H aliphatic stretching), 1662 (C=O stretching), 1564 (C=N stretching), 1467, 1393, 1333 (C=C aromatic ring stretching), 1428 (C-H aliphatic bending), 1113 (C-O stretching), 1053-761 (characteristic of thiazole); ^1^H-NMR (400 MHz, DMSO-d_6_): *δ* (ppm) 8.39-8.31 (m, 2H, Ar-H), 8.09 (d, 1H, Ar-H, *J* = 8.5 Hz), 8.03 (s, 1H, H-C=N), 4.21 (q, 2H, -CH_2_-CH_3_, *J* = 7.1 Hz), 2.48 (s, 3H, -CH_3_), 1.27 (t, 3H, CH_3_-CH_2_, *J* = 7.1 Hz); ^13^C-NMR (100 MHz, DMSO-d_6_): *δ* (ppm) 179.1 (Ar-C-CF_3_), 168.1 (thiazole C2), 163.6 (Ar-C-CF_3_), 162.2 (C=O), 159.1 (thiazole C4), 136.8 (C=N, azomethine), 136.3-122.4 (Ar-C), 119.7 (thiazole C5), 60.8 (CH_2_-O), 19.0 (-CH_3_), 14.7 (CH_3_-CH_2_-); HRMS: *m*/*z* calculated for C_16_H_13_F_6_N_3_O_2_S [M + H]^+^: calculated 426.0705, found 426.0696, [M + Na]^+^: calculated 448.0525, found 448.0506, [2M + Na]^+^: calculated 873.1158, found 873.1126.

***Ethyl (E)-2-(2-(2-hydroxy-3-methylbenzylidene)hydrazineyl)-4-methylthiazole-5-carboxylate* (3e)**. Light yellow solid; yield: 75%; melting point: 244–246 °C; R_f_: 0.54 (acetone/*n*-hexane, 1:3); FTIR (ATR) cm^−1^: 3157 (N-H stretching), 3054 (=C-H stretching), 2969 (C-H aliphatic stretching), 1629 (C=O stretching), 1568 (C=N stretching), 1533, 1483, 1374, 1329 (C=C aromatic ring stretching), 1432 (C-H aliphatic bending), 1101 (C-O stretching), 1039-745 (characteristic of thiazole); ^1^H-NMR (400 MHz, DMSO-d_6_): *δ* (ppm) 8.41 (s, 1H, H-C=N), 7.31 (d, 1H, Ar-H, *J* = 7.8 Hz), 7.19 (d, 1H, Ar-H, *J* = 6.9 Hz), 6.85 (t, 1H, Ar-H, *J* = 7.5 Hz), 4.21 (q, 2H, -CH_2_-CH_3_, *J* = 7.1 Hz), 2.45 (s, 3H, -CH_3_), 2.22 (s, 3H, -CH_3_), 1.27 (t, 3H, CH_3_-CH_2_, *J* = 7.1 Hz); ^13^C-NMR (100 MHz, DMSO-d_6_): *δ* (ppm) 167.4 (thiazole C2), 161.9 (C=O), 155.9 (thiazole C4), 132.7 (C=N, azomethine), 128.6, 125.4 119.8 (Ar-C), 118.4 (thiazole C5), 60.8 (CH_2_-O), 16.0 (CH_3_-), 14.8 (CH_3_-CH_2_); HRMS: *m*/*z* calculated for C_15_H_17_N_3_O_3_S [M + H]^+^: calculated 320.1063, found 320.1066, [M + Na]^+^: calculated 342.0883, found 342.0882, [2M + Na]^+^: calculated 661.1873, found 661.1857.

***Ethyl (E)-2-(2-(2,5-dimethylbenzylidene)hydrazineyl)-4-methylthiazole-5-carboxylate* (3f)**. Off-white solid; yield: 78%; melting point: 220–222 °C; R_f_: 0.69 (acetone/*n*-hexane, 1:3); FTIR (ATR) cm^−1^: 3204 (N-H stretching), 3103 (=C-H stretching), 2980 (C-H aliphatic stretching), 1654 (C=O stretching), 1560 (C=N stretching), 1426, 1395, 1364, 1325 (C=C aromatic ring stretching), 1094 (C-O stretching), 1043-724 (characteristic of thiazole); ^1^H-NMR (400 MHz, CDCl_3_): *δ* (ppm) 8.17 (s, 1H, H-C=N), 7.61 (s, 1H, Ar-H), 7.11 (d, 1H, Ar-H, *J* = 1.8 Hz), 4.30 (q, 2H, -CH_2_-CH_3_, *J* = 7.2 Hz), 2.65 (s, 3H, CH_3_), 2.45 (s, 3H, -CH_3_), 2.38 (s, 3H, -CH_3_) 1.39 (t, 3H, CH_3_-CH_2_, *J* = 7.2 Hz); ^13^C-NMR (100 MHz, CDCl_3_): *δ* (ppm) 170.3 (Thiazole C2), 162.6 (C=O), 156.9 (thiazole C4), 144.0 (C=N, azomethine), 135.8, 134.0, 131.3, 131.1, 130.8, 127.8 (Ar-C), 111.4 (thiazole C5), 60.7 (CH_2_-O), 21.0 (CH_3_-), 19.8 (-CH_3_), 17.3 (-CH_3_), 14.5 (CH_3_-CH_2_); HRMS: *m*/*z* calculated for C_16_H_19_N_3_O_2_S [M + H]^+^: calculated 318.1271, found 318.1265, [M + Na]^+^: calculated 340.1090, found 340.1080, [2M + Na] ^+^: calculated 657.2288, found 657.2261.

***Ethyl (E)-2-(2-(2,3-dimethoxybenzylidene)hydrazineyl)-4-methylthiazole-5-carboxylate* (3g)**. Gray solid; yield: 75%; melting point: 227–229 °C; R_f_: 0.64 (acetone/*n*-hexane, 1:3); FTIR (ATR) cm^−1^: 3150 (N-H stretching), 2943 (C-H aliphatic stretching), 1697 (C=O stretching), 1576 (C=N stretching), 1477, 1366, 1304 (C=C aromatic ring stretching), 1417 (C-H aliphatic bending), 1086 (C-O stretching), 1002-736 (characteristic of thiazole); ^1^H-NMR (400 MHz, CDCl_3_): *δ* (ppm) 8.33 (s, 1H, H-C=N), 7.60 (d, 1H, Ar-H, *J* = 6.5 Hz), 7.11 (t, 1H, Ar-H, *J* = 8.0 Hz), 6.96 (d, 1H, Ar-H, *J* = 6.5 Hz), 4.33 (q, 2H, -CH_2_-CH_3_, *J* = 7.2 Hz), 3.91 (s, 3H, -OCH_3_), 3.88 (s, 3H, -OCH_3_), 2.68 (s, 3H, -CH_3_), 1.39 (t, 3H, CH_3_-CH_2_, *J* = 7.2 Hz); ^13^C-NMR (100 MHz, CDCl_3_): *δ* (ppm) 170.5 (Thiazole C2), 162.7 (C=O), 157.6 (thiazole C4), 152.8 (Ar-C-OMe), 148.2 (Ar-C-OMe), 140.1 (C=N, azomethine), 127.4, 124.3, 117.8, 113.7 (Ar-C), 111.6 (thiazole C5), 61.6 (CH_2_-O), 60.7 (-OCH_3_), 55.9 (-OCH_3_), 17.3 (-CH_3_), 14.5 (CH_3_-CH_2_); HRMS: *m*/*z* calculated for C_16_H_19_N_3_O_4_S [M + H]^+^: calculated 350.1169, found 350.1165, [M + Na]^+^: calculated 372.0988, found 372.0980, [2M + Na]^+^: calculated 721.2085, found 721.2062.

***Ethyl (E)-2-(2-(2,4-dimethoxybenzylidene)hydrazineyl)-4-methylthiazole-5-carboxylate* (3h)**. Light brown solid; yield: 75%; melting point: 220–222 °C; R_f_: 0.60 (acetone/*n*-hexane, 1:3); FTIR (ATR) cm^−1^: 3179 (N-H stretching), 3064 (=C-H stretching), 2955 (C-H aliphatic stretching), 1691 (C=O stretching), 1586 (C=N stretching), 1533, 1502, 1370, 1313 (C=C aromatic ring stretching), 1428 (C-H aliphatic bending), 1127 (C-O stretching), 1031-757 (characteristic of thiazole); ^1^H-NMR (500 MHz, CDCl_3_): *δ* (ppm) 8.30 (s, 1H, H-C=N), 7.93 (d, 1H, Ar-H, *J* = 8.6 Hz), 6.57 (dd, 1H, Ar-H, *J* = 8.6, 2,4 Hz), 6.45 (d, 1H, Ar-H, *J* = 2.4 Hz), 4.31 (q, 2H, -CH_2_-CH_3_, *J* = 7.1 Hz), 3.87 (s, 3H, -OCH_3_), 3.83 (s, 3H, -OCH_3_), 2.65 (s, 3H, -CH_3_), 1.38 (t, 3H, CH_3_-CH_2_, *J* = 7.1 Hz); ^13^C-NMR (125 MHz, CDCl_3_): *δ* (ppm) 170.2 (Thiazole C2), 162.8 (C=O), 162.7 (Ar-C-OMe), 159.1 (thiazole C4), 157.6 (Ar-C-OMe), 140.7 (C=N, azomethine), 127.4, 115.2 (Ar-C), 110.9 (thiazole C5), 105.9, 97.9 (Ar-C), 60.6 (CH_2_-O), 55.5 (2x-OCH_3_) 17.1 (-CH_3_), 14.5 (CH_3_-CH_2_); HRMS: *m*/*z* calculated for C_16_H_19_N_3_O_4_S [M + H]^+^: calculated 350.1169, found 350.1164, [M + Na] ^+^: calculated 372.0988, found 372.0982, [2M + Na]^+^: calculated 721.2085, found 721.2070.

***Ethyl (E)-2-(2-(2,5-dimethoxybenzylidene)hydrazineyl)-4-methylthiazole-5-carboxylate* (3i)**. Beige solid; yield: 77%; melting point: 222–224 °C; R_f_: 0.66 (acetone/*n*-hexane, 1:3); FTIR (ATR) cm^−1^: 3045 (=C-H stretching), 2932 (C-H aliphatic stretching), 1697 (C=O stretching), 1576 (C=N stretching), 1537, 1494, 1368 (C=C aromatic ring stretching), 1422 (C-H aliphatic bending), 1078 (C-O stretching), 1022-794 (characteristic of thiazole); ^1^H-NMR (500MHz, CDCl_3_): *δ* (ppm) 8.35 (s, 1H, H-C=N), 7.52 (d, 1H, Ar-H, *J* = 3.1 Hz), 6.93 (dd, 1H, Ar-H, *J* = 8.9, 3.1 Hz), 6.85 (d, 1H, Ar-H, *J* = 8.9 Hz) 4.30 (q, 2H, -CH_2_-CH_3_, *J* = 7.1 Hz), 3.87 (s, 3H, -OCH_3_), 3.81 (s, 3H, -OCH_3_), 2.65 (s, 3H, -CH_3_), 1.38 (t, 3H, CH_3_-CH_2_, *J* = 7.1 Hz); ^13^C-NMR (125 MHz, CDCl_3_): *δ* (ppm) 170.7 (thiazole C2), 162.8 (C=O), 157.9 (thiazole C4), 153.7 (C=N, azomethine), 152.4, 140.1, 122.8, 117.4, 112.4, 111.2 (Ar-C), 110.2 (thiazole C5), 60.6 (CH_2_-O), 56.0 (-OCH_3_), 55.9 (-OCH_3_), 17.1 (CH_3_-), 14.5 (CH_3_-CH_2_); HRMS: *m*/*z* calculated for C_16_H_19_N_3_O_4_S [M + H]^+^: calculated 350.1169, found 350.1160, [M + Na]^+^: calculated 372.0988, found 372.0975, [2M + Na]^+^: calculated 721.2085, found 721.2064.

***Ethyl (E)-2-(2-(3,5-dimethoxybenzylidene)hydrazineyl)-4-methylthiazole-5-carboxylate* (3j)**. Off-white solid; yield: 77%; melting point: 206–208 °C; R_f_: 0.66 (acetone/*n*-hexane, 1:3); FTIR (ATR) cm^−1^: 3212 (N-H stretching), 3099 (=C-H stretching), 2965 (C-H aliphatic stretching), 1669 (C=O stretching), 1562 (C=N stretching), 1525, 1463, 1364, 1317 (C=C aromatic ring stretching), 1415 (C-H aliphatic bending), 1121 (C-O stretching), 1086-726 (characteristic of thiazole); ^1^H-NMR (500 MHz, CDCl_3_): *δ* (ppm) 7.85 (s, 1H, H-C=N), 6.83 (d, 2H, Ar-H, *J* = 2.2 Hz), 6.52 (t, 1H, Ar-H, *J* = 2.2 Hz), 4.32 (q, 2H, -CH_2_-CH_3_, *J* = 7.1 Hz), 3.86 (s, 6H, 2X-OCH_3_), 2.63 (s, 3H, -CH_3_), 1.39 (t, 3H, CH_3_-CH_2_, *J* = 7.1 Hz); ^13^C-NMR (125 MHz, CDCl_3_): *δ* (ppm) 169.8 (Thiazole C2), 162.5 (C=O), 161.0 (Ar-C-OMe), 156.6 (thiazole C4), 144.5 (C=N, azomethine), 135.4 (Ar-C), 111.7 (thiazole C5), 105.0, 102.6 (Ar-C), 60.8 (CH_2_-O), 55.5 (2x-OCH_3_) 17.1 (-CH_3_), 14.4 (CH_3_-CH_2_); HRMS: *m*/*z* calculated for C_16_H_19_N_3_O_4_S [M + H]^+^: calculated 350.1169, found 350.1161, [M + Na]^+^: calculated 372.0988, found 372.0977, [2M + Na]^+^: calculated 721.2085, found 721.2064.

***Ethyl (E)-4-methyl-2-(2-(2,4,5-trimethoxybenzylidene)hydrazineyl)thiazole-5-carboxylate* (3k)**. Light purple solid; yield: 72%; melting point: 234–235 °C; R_f_: 0.59 (acetone/*n*-hexane, 1:3); FTIR (ATR) cm^−1^: 3140 (N-H stretching), 2936 (C-H aliphatic stretching), 1695 (C=O stretching), 1599 (C=N stretching), 1572, 1516, 1370, 1310 (C=C aromatic ring stretching), 1420 (C-H aliphatic bending), 1072 (C-O stretching), 1024-755 (characteristic of thiazole); ^1^H-NMR (400 MHz, CDCl_3_): *δ* (ppm) 8.31 (s, 1H, H-C=N), 7.48 (s, 1H, Ar-H), 6.48 (s, 1H, Ar-H), 4.30 (q, 2H, -CH_2_-CH_3_, *J* = 7.1 Hz), 3.96 (s, 3H, -OCH_3_), 3.94 (s, 3H, -OCH_3_), 3.83 (s, 3H, -OCH_3_), 2.64 (s, 3H, -CH_3_), 1.37 (t, 3H, CH_3_-CH_2_, *J* = 7.1 Hz); ^13^C-NMR (100 MHz, CDCl_3_): *δ* (ppm) 170.5 (Thiazole C2), 162.9 (C=O), 158.2 (thiazole C4), 153.3 (Ar-C-OMe), 152.1 (Ar-C-OMe), 143.7 (Ar-C-OMe), 140.3 (C=N, azomethine), 113.9 (Ar-C), 110.9 (thiazole C5), 108.2, 96.6 (Ar-C), 60.5 (CH_2_-O), 56.5 (-OCH_3_), 56.3 (-OCH_3_), 56.1 (-OCH_3_), 17.3 (-CH_3_), 14.5 (CH_3_-CH_2_); HRMS: *m*/*z* calculated for C_17_H_21_N_3_O_5_S [M + H]^+^: calculated 380.1275, found 380.1276, [M + Na]^+^: calculated 402.1094, found 402.1093, [2M + Na]^+^: calculated 781.2296, found 781.2283.

***Ethyl (E)-2-(2-((4,5-dimethylthiazol-2-yl)methylene)hydrazineyl)-4-methylthiazole-5-carboxylate* (3l)**. Yellow solid; yield: 66%; melting point: 219–221 °C; R_f_: 0.51 (acetone/*n*-hexane, 1:3); FTIR (ATR) cm^−1^: 3152 (N-H stretching), 3039 (=C-H stretching), 2916 (C-H aliphatic stretching), 1605 (C=O, ester stretching), 1564 (C=N stretching), 1531, 1426, 1376, 1356 (C=C aromatic ring stretching), 1111 (C-O stretching), 1024-740 (characteristic of thiazole); ^1^H-NMR (500 MHz, DMSO-d_6_): *δ* (ppm) 8.10 (s, 1H, H-C=N), 4.21 (q, 2H, O-CH_2_-CH_3_, *J* = 7.1 Hz) 2.47 (s, 3H, CH_3_-), 2.36 (s, 3H, H_3_C-thiazole-C5), 2.27 (s, 3H, H_3_C-thiazole-C4), 1.27 (t, 3H, CH_3_-CH_2_, *J* = 7.1 Hz); ^13^C-NMR (125 MHz, DMSO-d_6_): *δ* (ppm) 169.0 (thiazole ring C2), 162.2 (C=O), 159.2 (thiazole C2), 155.2 (thiazole C4), 149.9 (thiazole C4), 147.1 (HC=N- azomethine), 142.4 (thiazole C5), 128.9 (thiazole C5), 60.8 (CH_2_-O), 14.9 (CH_3_-) 14.7 (CH_3_-), 14.6 (CH_3_-), 11.7 (CH_3_-CH_2_-); HRMS: *m*/*z* calculated for C_13_H_16_N_4_O_2_S_2_, [M + H]^+^: 325.0787, found 325.0781, [M + Na]^+^: calculated 347.0607, found 347.0604, [2M + Na]^+^: calculated 671.1322, found 671.1305.

***Ethyl (E)-2-(2-((3-bromothiophen-2-yl)methylene)hydrazineyl)-4-methylthiazole-5-carboxylate* (3m)**. Light yellow solid; yield: 72%; melting point: 210–212 °C; R_f_: 0.56 (acetone/*n*-hexane, 1:3); ^1^H-NMR (400MHz, DSMO-d_6_): *δ* (ppm) 8.23 (s, 1H, H-C=N-), 7.75 (d, 1H, thiophene-H, *J* = 4.6 Hz), 7.19 (d, 1H, thiophene-H, *J* = 5.4 Hz), 4.21 (q, 2H, -CH_2_-CH_3_, *J* = 7.2 Hz), 2.47 (s, 3H, -CH_3_), 1.27 (t, 3H, CH_3_-CH_2_, *J* = 7.2 Hz); ^13^C-NMR (100 MHz, DMSO-d_6_): *δ* (ppm) 168.9 (Thiazole C2), 162.2 (C=O), 133.8 (H-C=N- azomethine) 131.3 (thiophene C5), 131.2 (thiophene C4), 130.2 (thiophene C2), 129.9 (thiophene C3), 113.0 (thiazole C5), 60.7 (CH_2_-O-), 17.3 (CH_3_-), 14.8 (CH_3_-CH_2_); HRMS: *m*/*z* calculated for C_12_H_12_BrN_3_O_2_S_2_ [M + H]^+^: calculated 373.9627, found 373.9627, [M + Na]^+^: calculated 395.9447, found 395.9441, [2M + Na]^+^: calculated 768.9001, found 768.8985.

### 3.2. Biological Evaluation

#### 3.2.1. Cytotoxicity

A suspension containing 10^4^ NIH/3T3 cells (ATCC, catalog number CRL-1658, USA) in RPMI-1240 medium (Sigma-Aldrich, St. Louis, MO, USA) supplemented with 10% (*v*/*v*) fetal bovine serum (Cultilab, Campinas, SP, Brazil) and antibiotics (100 units penicillin and 0.01 mg streptomycin/mL, Sigma-Aldrich, USA) was plated in 96-well plates and allowed to adhere, and then it was incubated at 37 °C in a 5% CO_2_ incubator (ThermoFischer, Waltham, MA, USA) for 18 h. Supernatants were replaced with the supplemented RPMI medium containing the compounds at concentrations ranging from 250 to 15.6 μM in quadruplicates. The plates were then incubated at 37 °C and 5% CO_2_ for 48 h, after which viability was assessed through the resazurin method: 5 µL of a 0.2 mg/mL (*m*/*v*) resazurin solution (Sigma-Aldrich, USA) was added to each well and, after 4 h incubation, absorbance was read at 570 and 600 nm with plate reader spectrophotometer (SpectraMax, Molecular Devices, San Jose, CA, USA). Negative control wells containing only medium and positive controls containing amphotericin B (50 to 0.78 µM, Sigma-Aldrich, USA) and pentamidine (250 to 7.8 µM, Sigma-Aldrich, USA) were also evaluated. The percentage of viability for each compound concentration was calculated compared to the live control with medium alone, considered 100% live cells.

#### 3.2.2. Antipromastigote Activity

Promastigote forms (2 × 10^5^) of *L.* (L.) *amazonensis* PH8 strain (IFLA/BR/1967/PH8, code 0575 from CLIOC Leishmania Collection, Oswaldo Cruz Institute, Rio de Janeiro – RJ, Brazil) in Schneider’s Insect medium (Sigma-Aldrich, USA) supplemented with 20% (*v*/*v*) fetal bovine serum (Cultilab, Campinas, SP, Brazil) and antibiotics (100 units penicillin and 0.01 mg streptomycin/mL, Sigma-Aldrich, USA) were distributed in 96-well plates containing the compounds at concentrations ranging from 25 to 1.56 μM in quadruplicate. The plates were incubated at 26 °C for 48 h (BOD incubator, Marqlabor, Matão, SP, Brazil), after which viability was assessed spectrophotometrically using the resazurin method (Sigma-Aldrich, USA) as described above. Negative control containing only medium and positive controls containing amphotericin B (5 to 0.078 µM, Sigma-Aldrich, USA) and pentamidine (250 to 3.90 µM, Sigma-Aldrich, USA) were also evaluated, as reference antileishmanial drugs. The percentage of viability for each compound concentration was calculated in relation to the negative control with medium alone, which was considered to represent 100% live cells.

#### 3.2.3. Antiamastigote Activity

The methodology for assessing antileishmanial activity against intracellular amastigotes was adapted [85] using murine peritoneal macrophages. This protocol was approved by the Animal Ethics Committee (CEUA) of Federal University of Mato Grosso do Sul (UFMS) under certificate number 1.041/2019 and Balb/c female mice were provided by Central Animal Facility from Institute of Biosciences, UFMS on 23 April 2019, and ran through 31 July 2024. Peritoneal macrophages were collected after the animals were anesthetized and euthanized. The 96-well microplates were seeded with murine peritoneal macrophages (10^5^) in supplemented RPMI medium and incubated at 37 °C with 5% CO_2_ for 1 h to allow adhesion. Adherent macrophages were infected with promastigote forms of *L. amazonensis* in the stationary phase (7-day culture) and then incubated at 35 °C/5% CO_2_ for 4 h to allow macrophage infection. After this period, the wells were washed with phosphate-buffered solution (PBS) and infected macrophages were then treated for 48 h with the compounds at concentrations ranging from 50 to 0.78 µM. Wells containing infected, untreated macrophages were used as a negative infection control, and wells treated with amphotericin B (1 to 0.0156 µM, Sigma-Aldrich, USA) and pentamidine (10 to 0.156 µM, Sigma-Aldrich, USA) were used as positive control, as reference antileishmanial drugs. After 48 h of incubation, the supernatants were removed, cells were washed with PBS, 150 µL of supplemented Schneider’s medium was added to each well, and the plates were incubated at 26 °C for 5 days to allow viable intracellular amastigotes to transform into promastigotes and multiply. Then, the viability of the promastigote forms in the microplates was assessed using the resazurin method, as described above.

#### 3.2.4. Statistical Analysis

Experiments were conducted in quadruplicate. The one-way ANOVA test and Tukey’s post hoc test were used to compare variances, and the IC_50_ and CC_50_ values of the compounds were obtained through nonlinear regression based on the viability results. All graphs and analyses were performed using GraphPad Prism 5.0 software, assuming a 95% confidence interval (GraphPad Software, San Diego, CA, USA).

### 3.3. Molecular Docking

Molecular docking studies were performed targeting Leishmania trypanothione synthetase using the crystal structure with PDB ID: 2VOB obtained from the RCSB Protein Data Bank. The 3D structures of the antioxidant compounds **3b**, **3c**, **3h**, **3i**, **3k** and **3m** were generated using the Avogadro molecular editor and subsequently optimized by applying the MMFF94 force field. These optimized molecules were then converted into PDBQT format for docking studies. For protein preparation, water molecules were removed, polar hydrogens were added, and Kollman charges were assigned using AutoDock Tools. A grid box measuring 116 × 126 × 110 Å was set to cover the active site and allow ligand flexibility, with the center coordinates fixed at (x: −34.103, y: −8.467, z: 30.552) and a grid spacing of 0.667 Å, based on the protein’s catalytic region. During docking, the ligands were considered flexible, while the protein structure remained rigid. Docking simulations were carried out using the Lamarckian Genetic Algorithm (LGA) embedded in AutoDock 4.2, employing a population size of 150, a maximum of 2,500,000 energy evaluations, and 100 GA runs per ligand. Visualization and analysis of docking results were performed using BIOVIA Discovery Studio Visualizer, Version 21.1.0.20298, Dassault Systèmes BIOVIA, San Diego, CA, USA.

## 4. Conclusions

In summary, a series of novel arylidenehydrazinyl 4-methylthiazole-5-carboxylate derivatives was synthesized and evaluated as preliminary hits against *Leishmania amazonensis*. The most active compound of the series, **3m**, displayed moderate antiamastigote activity and molecular docking studies provided supporting evidence for interaction with *Leishmania* trypanothione synthetase as a plausible mechanism of action. Although the biological potency of the present compounds remains modest in comparison with reference drugs, these results represent an encouraging starting point for the rational design of new synthetic molecules. Structural optimization guided by the pharmacological and molecular data reported herein may contribute to the development of more potent and selective antileishmanial candidates targeting trypanothione synthetase.

## Figures and Tables

**Table 1:** Synthesized arylidenehydrazinyl 4-methylthiazole-5-carboxylates derivatives **3a**–**m**.

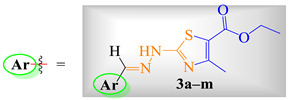		
Compound	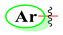	Yield %
**3a**	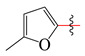	72
**3b**	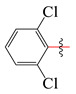	75
**3c**	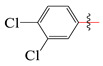	73
**3d**	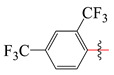	71
**3e**	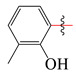	75
**3f**	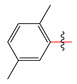	78
**3g**	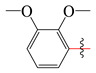	75
**3h**	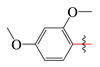	75
**3i**	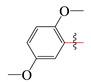	77
**3j**	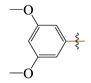	77
**3k**	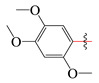	72
**3l**	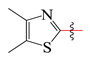	66
**3m**	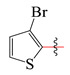	72

**Table 2:** Antiamastigote activity, cytotoxicity and selectivity of compounds **3m**, **3c**, **3h** and **3b**.

Compound	CC_50_ NIH/3T3 (µM ± SD)	IC_50_ Promastigotes (µM ± SD)	IC_50_ Amastigotes (µM ± SD)	SI (Promastigotes)	SI (Amastigotes)
**3a**	>250	19.76 ± 1.2	>50	>12.6	nd
**3b**	>250	>25	34.18 ± 1.2	nd	>7.3
**3c**	>250	>25	32.86 ± 1.1	nd	>7.6
**3d**	56.96 ± 1.6	>25	>50	1.8	nd
**3e**	102.1	>25	>50	nd	nd
**3f**	>250	>25	>50	nd	nd
**3g**	>250	>25	>50	nd	nd
**3h**	>250	>25	33.64 ± 1.2	nd	>7.4
**3i**	>250	>25	>50	nd	nd
**3j**	>250	>25	>50	nd	nd
**3k**	>250	>25	>50	nd	nd
**3l**	>250	>25	>50	nd	nd
**3m**	120.7 ± 1.4	>25	15.92 ± 1.1	nd	7.6
**ANFB**	10.48 ± 1.2	0.18 ± 0.07	0.26 ± 0.08	58.2	40.3
**pentamidine**	23.61 ± 1.8	2.05 ± 1.3	0.61 ± 0.09	11.5	38.7

CC_50_ = cytotoxic concentration against NIH/3T3 cells; IC_50_ = 50% inhibitory concentration against parasites; SD = standard deviation; SI promastigotes = selectivity index between promastigote forms and NIH/3T3; SI amastigotes = selectivity index between amastigote forms and NIH/3T3. ANFB = amphotericin B; nd = not determined.

**Table 3:** Binding energies, and number of hydrogen bonding for antioxidants **3b**, **3c**, **3h**, **3i**, **3k**, **3m** ^[a]^.

Compound	Binding Energy (kcal/mol)	Inhibition Constant (µM)	Amino Acid Residue Involved in H-Bonding, Alkyl/II-Alkyl Interactions
**3b**	−4.11	27.12	Ser264, Trp363
**3c**	−5.28	21.57	His543, Lys542, Glu587
**3h**	−5.03	31.08	Thr641, Gln86, Lys643, Gln103
**3i**	−3.16	41.68	Gun86, Gln103
**3k**	−5.14	29.13	Thr641, Leu86, Val101, Gln103
**3m**	−5.37	18.47	Thr337, Gly338, Lys217, Arg45
**Pentamidine**	−2.96	10.21	Pro215, Arg275

^[a]^ 2VOB enzyme as target protein was taken. The inhibition constant (K_i_) values were estimated automatically by AutoDock 4.2 based on the predicted binding free energies using the standard thermodynamic relationship between binding energy and inhibition constant at 298 K.

## Data Availability

The original contributions presented in this study are included in the article and Supplementary Materials. The datasets used and/or analyzed during the current study are available from the corresponding authors upon request.

## Supplementary Materials

The following supporting information can be downloaded at: https://www.mdpi.com/article/10.3390/molecules31132278/s1, ^1^HNMR and ^13^CNMR—Figures S1–S27; HRMS—Figures S28–S40; IR—Figures S41–S52; Binding model of compounds—Figures S53 and S54; Biological Activity of compounds **3a**–**m** and for reference drugs **amphotericin B** and **pentamidine**—Figures S56–S58.
