# Supplementary material for: Arylidenehydrazinyl 4-Methylthiazole-5-carboxylates: Synthesis, Antileishmanial Activity, and Targeting of Trypanothione Synthetase
Source: Molecules. 2026 Jun 29;31(13):2278. doi: 10.3390/molecules31132278 (PMC13362754; doi:10.3390/molecules31132278)

Supporting Information

Arylidenehydrazinyl 4-Methylthiazole-5-Carboxylates: Synthesis, Antileishmanial Activity, and Targeting of Trypanothione Synthetase

Brunno da S. Souza [1,†], Hasnain Mehmood [2,†], Estela M. Nolasco [3], Muhammad Haroon [2,4], Tashfeen Akhtar [2], Nathalia da Silva Brito [1], Adilson Beatriz [5], Nikhil Sodhi [6], Vijay P. Singh [6], Amilcar M. Junior [7], Gleison A. Casagrande [5], Dênis Pires de Lima [5], Sumbal Saba [1]*, Thalita B. Riul [3,]* and Jamal Rafique [1,5,]*

[1] Laboratório de Síntese Sustentável e Organocalcogênio (LabSO), Instituto de Química (IQ), Universidade Federal de Goiás (UFG), Goiânia 74690-900, GO, Brazil;

[2] Department of Chemistry, Mirpur University of Science and Technology (MUST), Mirpur 10250, AJK, Pakistan;

[3] Faculdade de Ciências Farmacêuticas, Alimentos e Nutrição (FACFAN), Universidade Federal do Mato Grosso do Sul (UFMS), Campo Grande 79074-460, MS, Brazil;

[4] Department of Chemistry, Government Degree College, Mirpur 10250, AJK, Pakistan;

[5] Instituto de Química (INQUI), Universidade Federal do Mato Grosso do Sul (UFMS), Campo Grande 79074-460, MS, Brazil;

[6] Department of Chemistry & Centre of Advanced Studies in Chemistry, Panjab University, Chandigarh 160014, Punjab, India;

[7] Instituto de Química, Universidade Federal do Rio Grande do Norte (UFRN), Av. Senador Salgado Filho, s/n, Natal 59078-970, RN, Brazil;

[†] These authors contributed equally to this work.

*Correspondence: sumbalsaba@ufg.br (S.S.); thalita.riul@ufms.br (T.B.R.); jamal.chm@gmail.com or jamal.rafique@ufms.br (J.R.)

Table of Contents

I. ^{1}H NMR and ^{13}C NMR Spectra of compounds 3a-m S2
II. Mass Spectrum of compounds 3a-m S16
III. IR Spectrum of compounds 3a-m S22
IV. 3D and 2D-diagram of compounds 3b, 3c, 3h, 3i, 3k, 3m. *Leishmania* trypanothione synthetase.. S29
V. Biological Activity of compounds 3a-m... S31

^{1}H NMR and ^{13}C NMR Spectra of compounds 3a-m

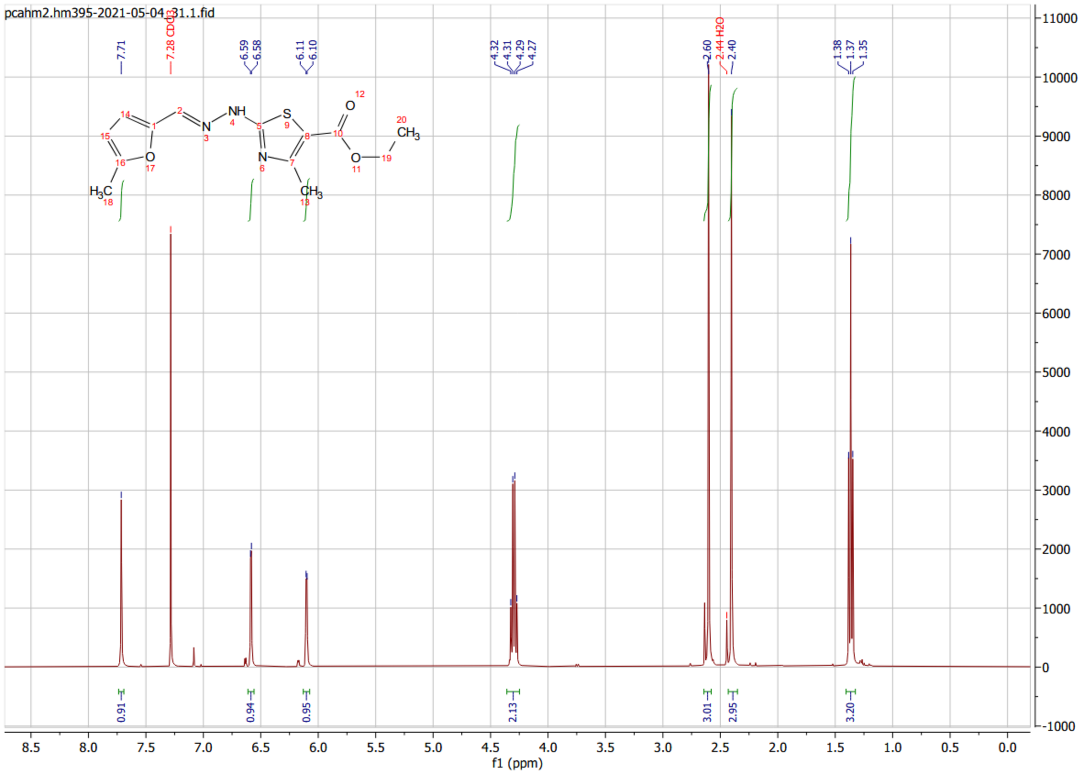

Figure S1. ^{1}H NMR Spectrum of 3**a**

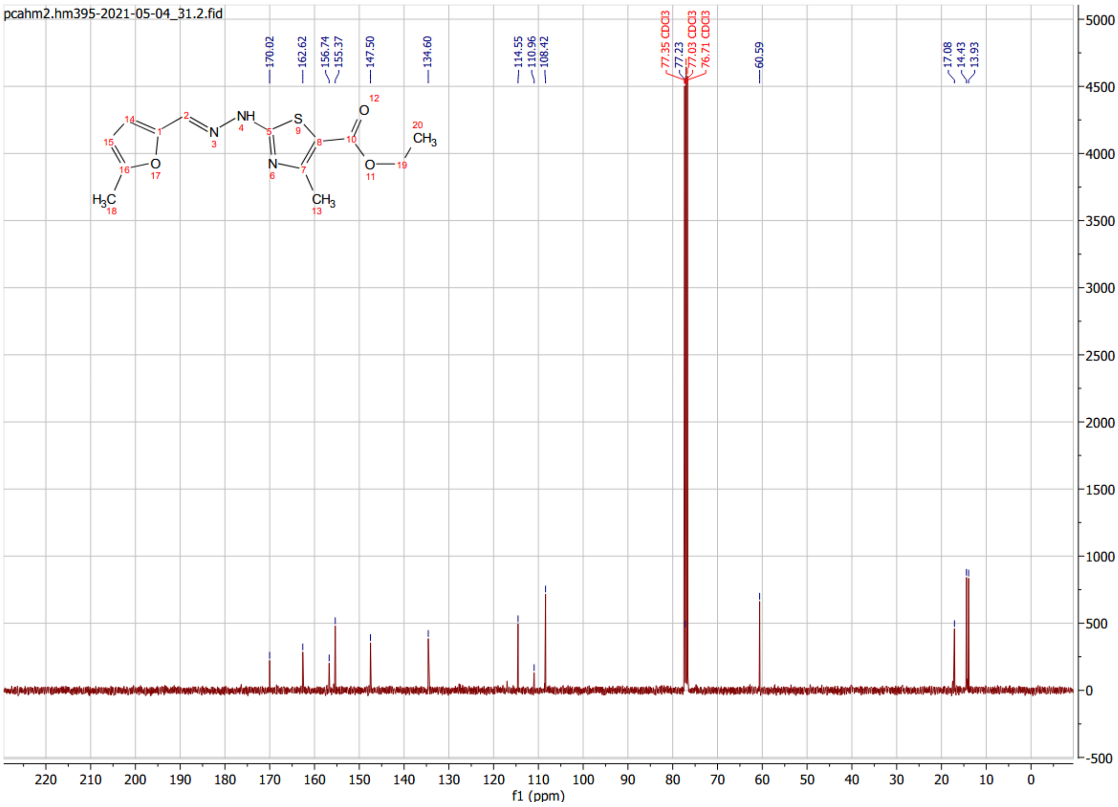

Figure S2. ^{13}C NMR Spectrum of **3a**

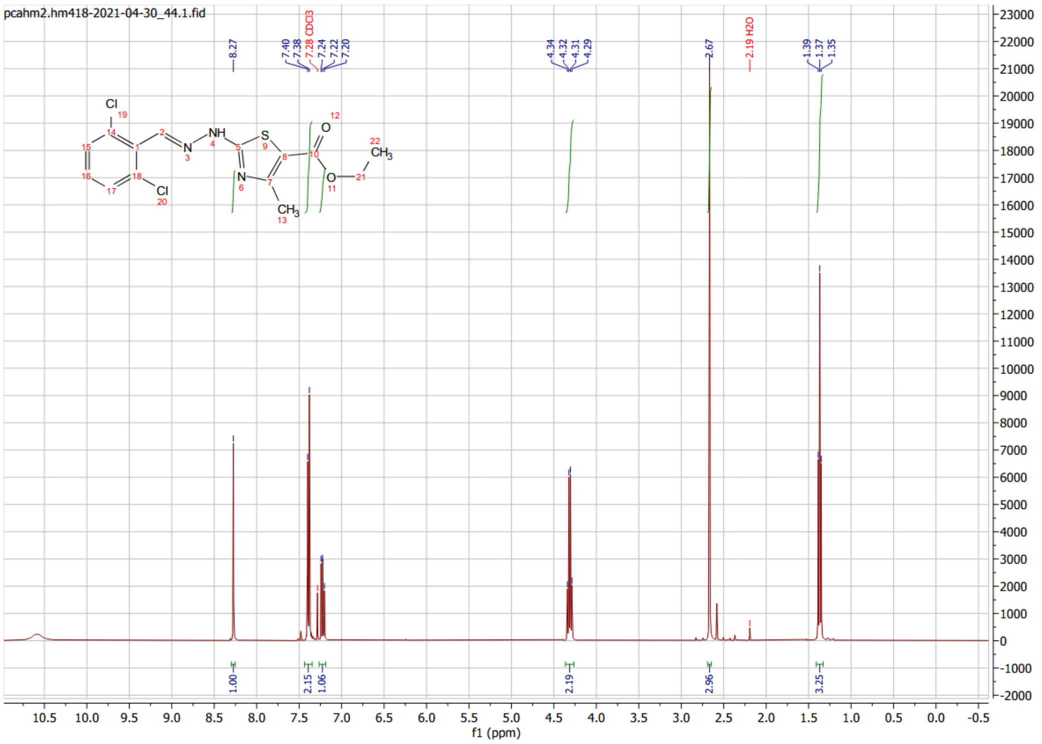

Figure S3. ^{1}H NMR Spectrum of **3b**

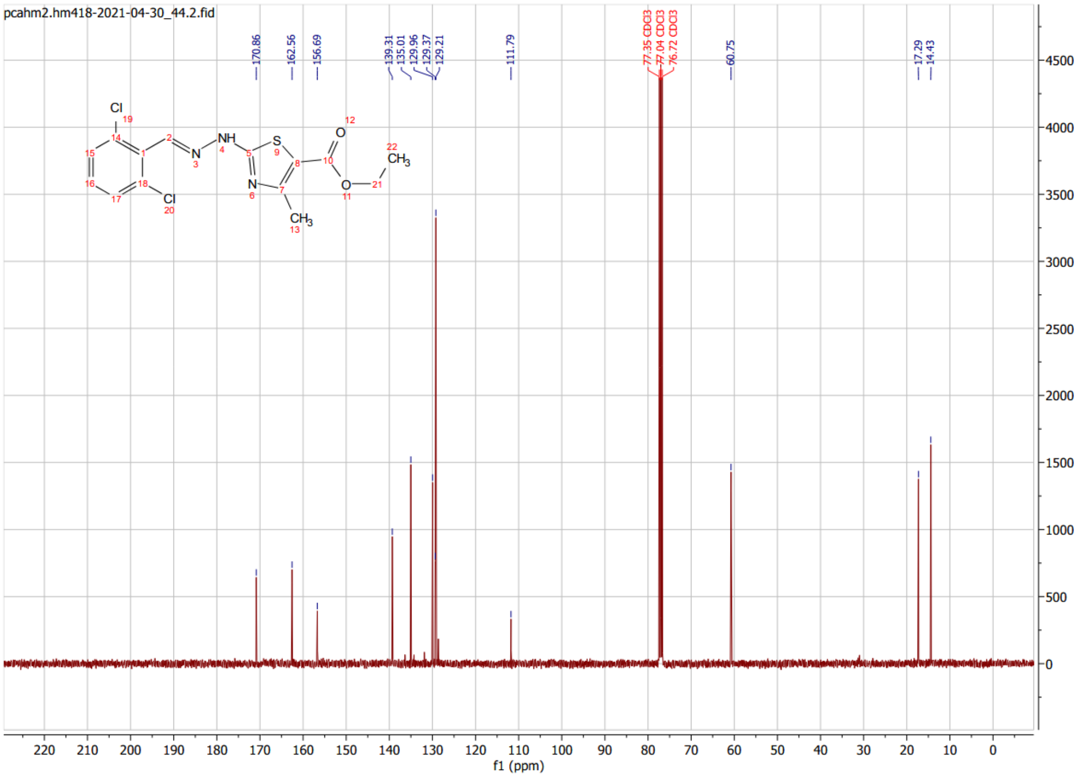

Figure S4. ^{13}C NMR Spectrum of **3b**

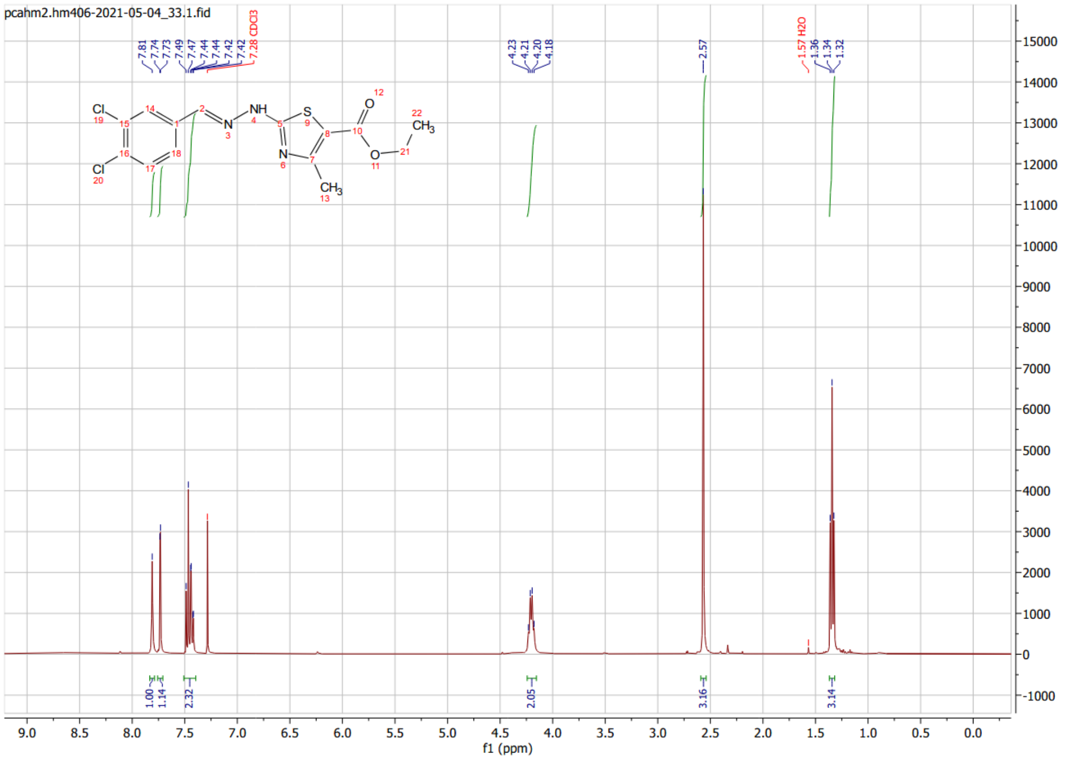

Figure S5. ^{1}H NMR Spectrum of **3c**

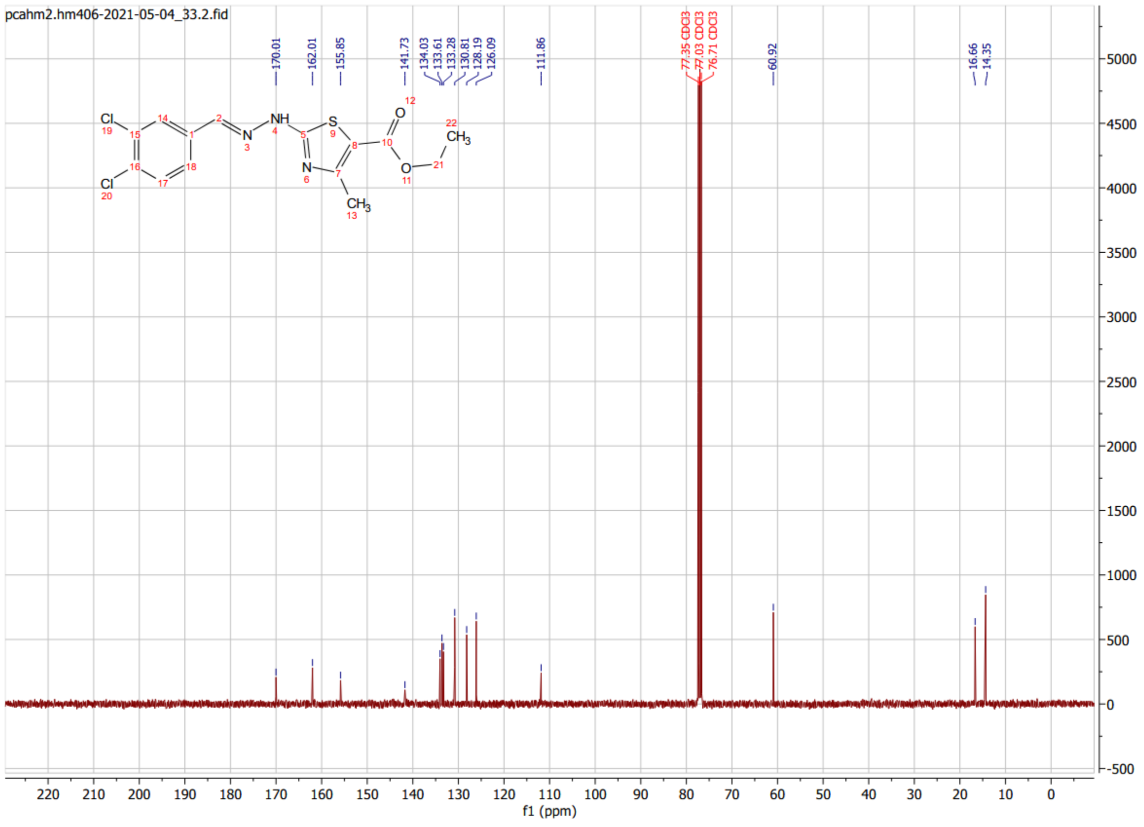

Figure S6. ^{13}C NMR Spectrum of **3c**

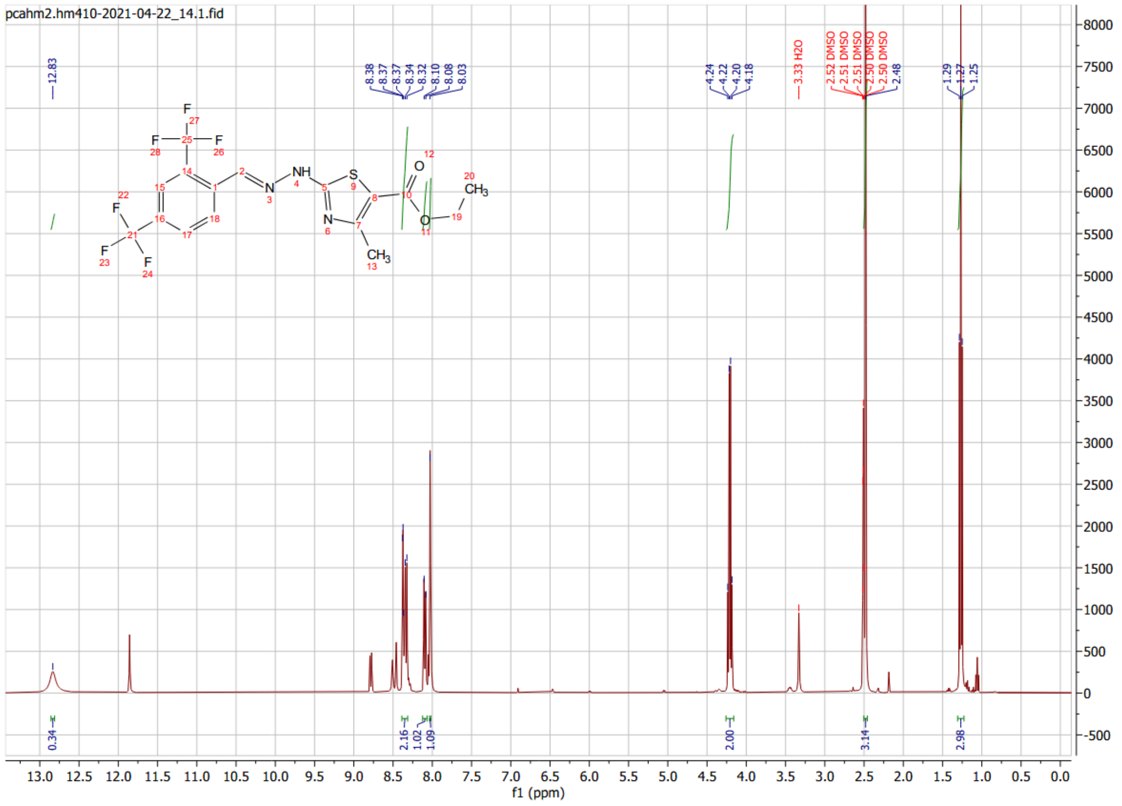

Figure S7. ^{1}H NMR Spectrum of **3d**

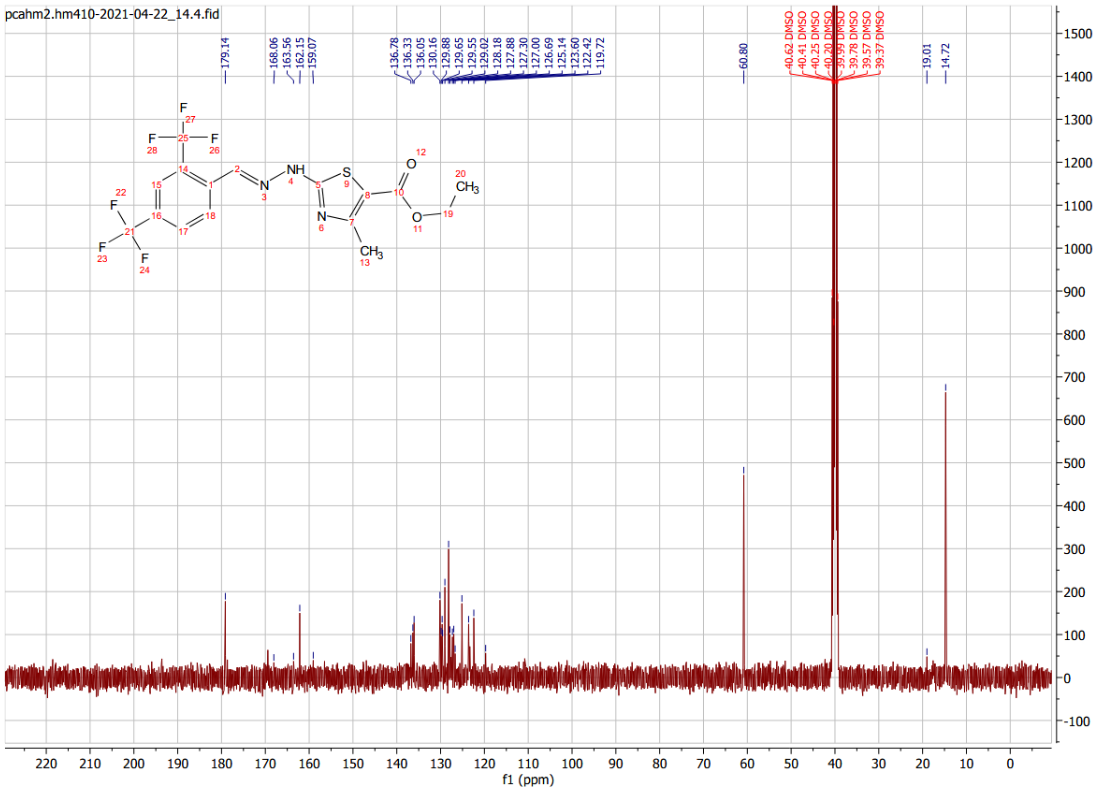

Figure S8. ^{13}C NMR Spectrum of **3d**

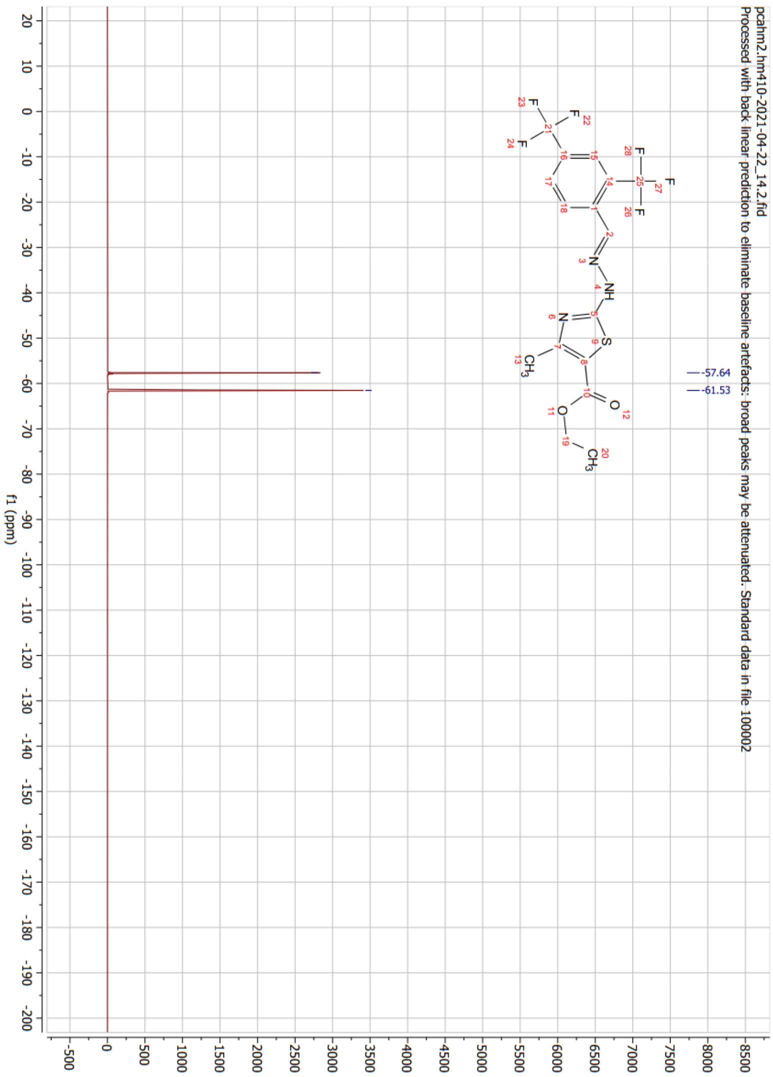

Figure S9. ^{19}F NMR Spectrum of **3d**

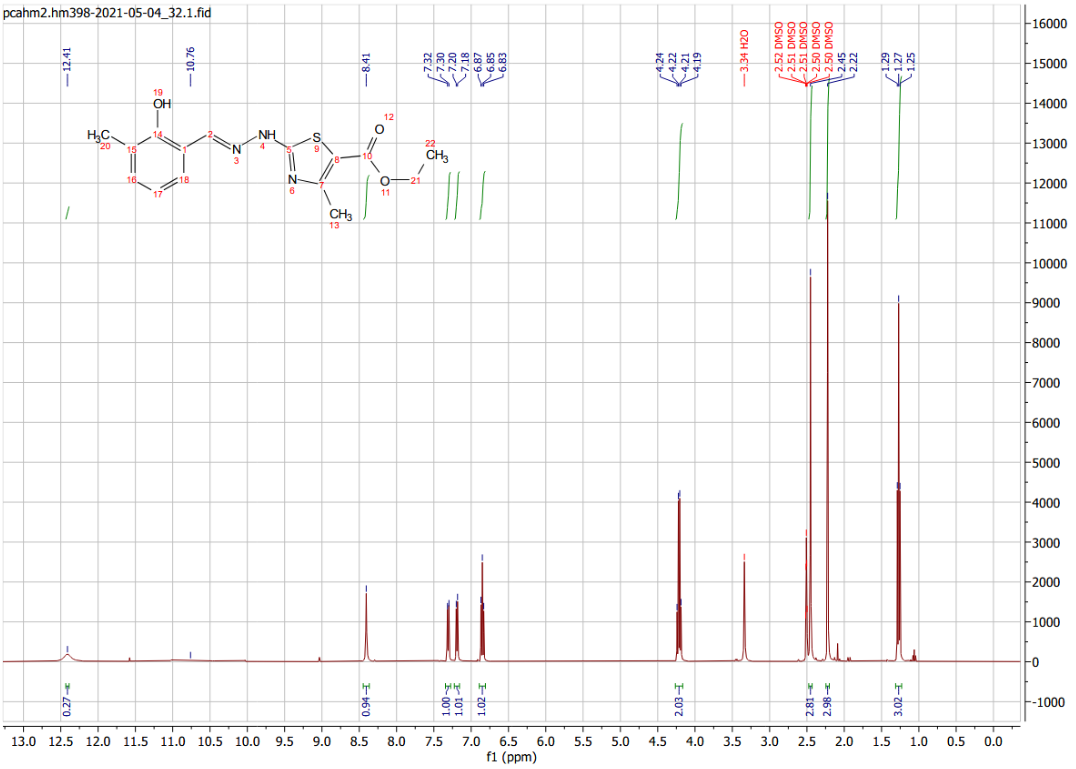

Figure S10. ^{1}H NMR Spectrum of **3e**

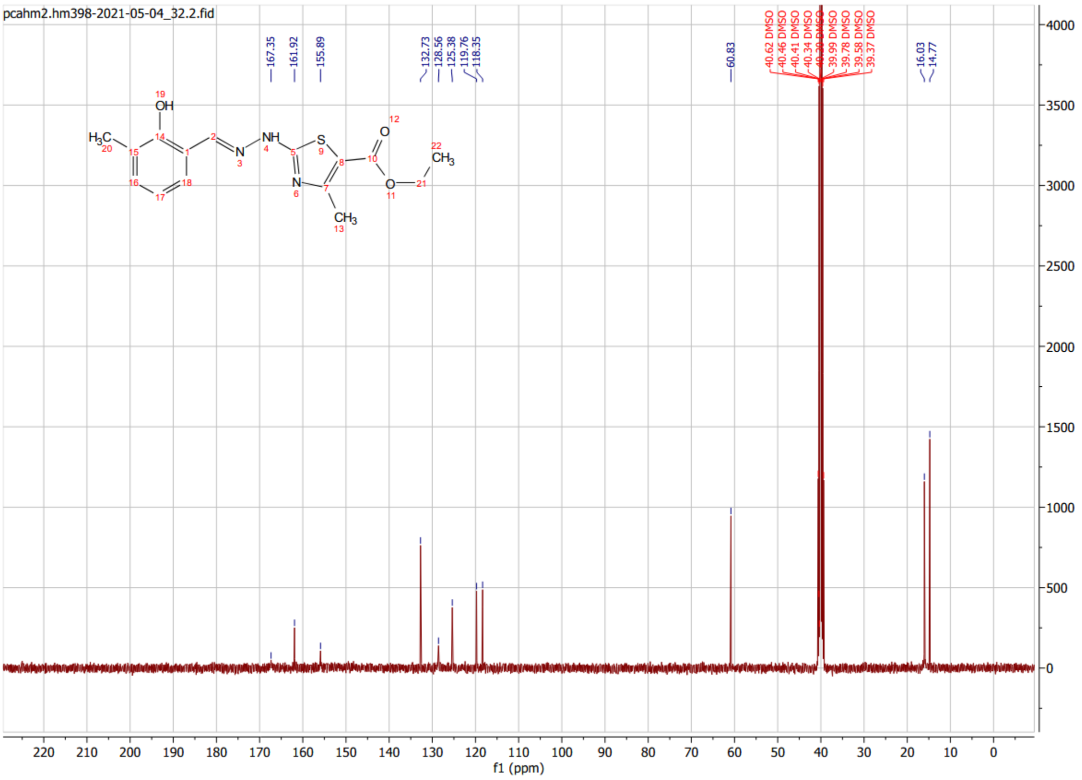

Figure S11. ^{13}C NMR Spectrum of **3e**

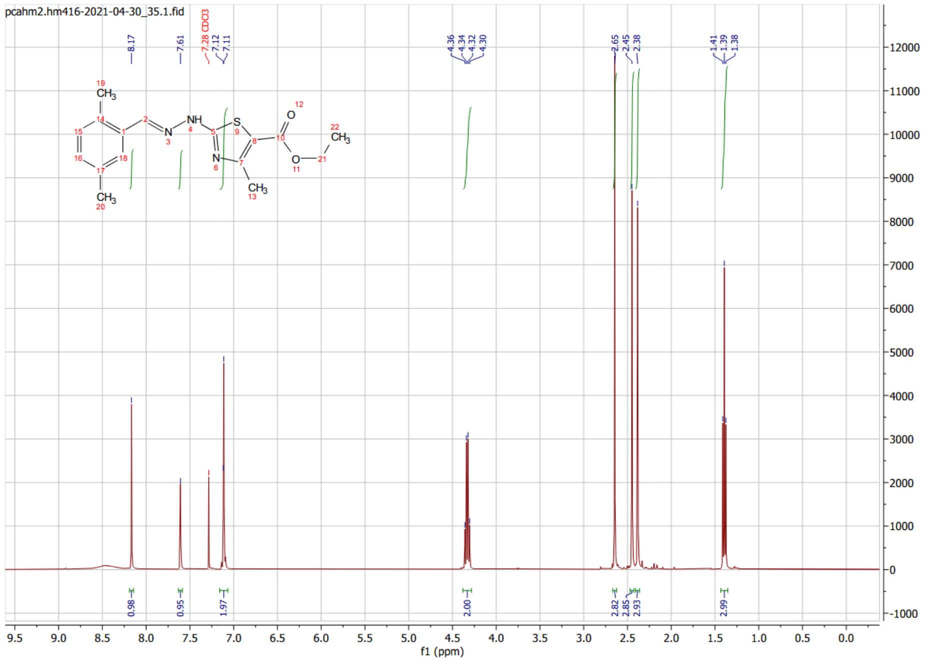

Figure S12. ^{1}H NMR Spectrum of **3f**

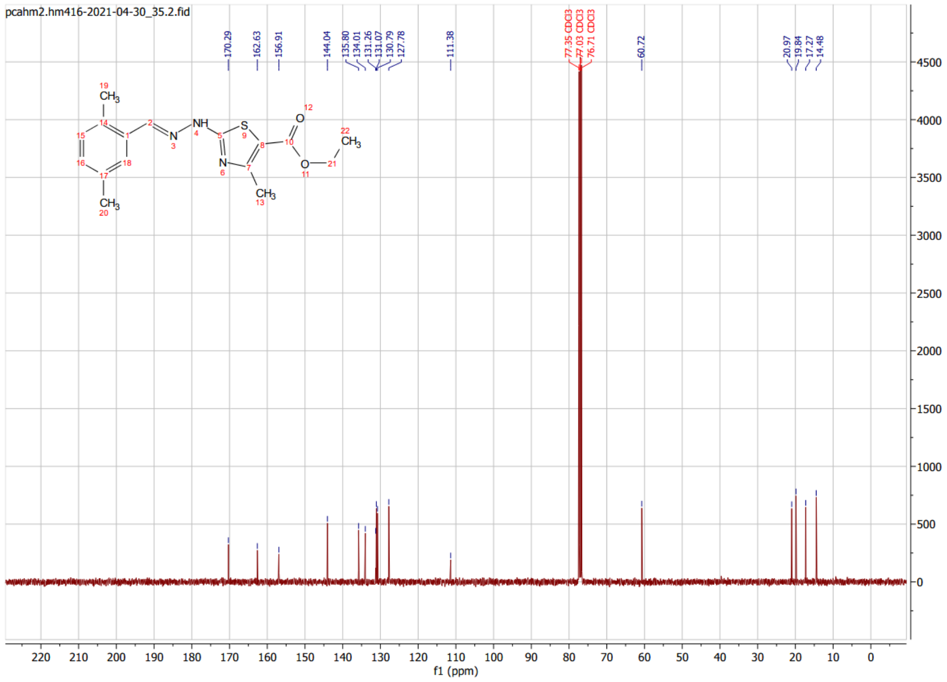

Figure S13. ^{13}C NMR Spectrum of **3f**

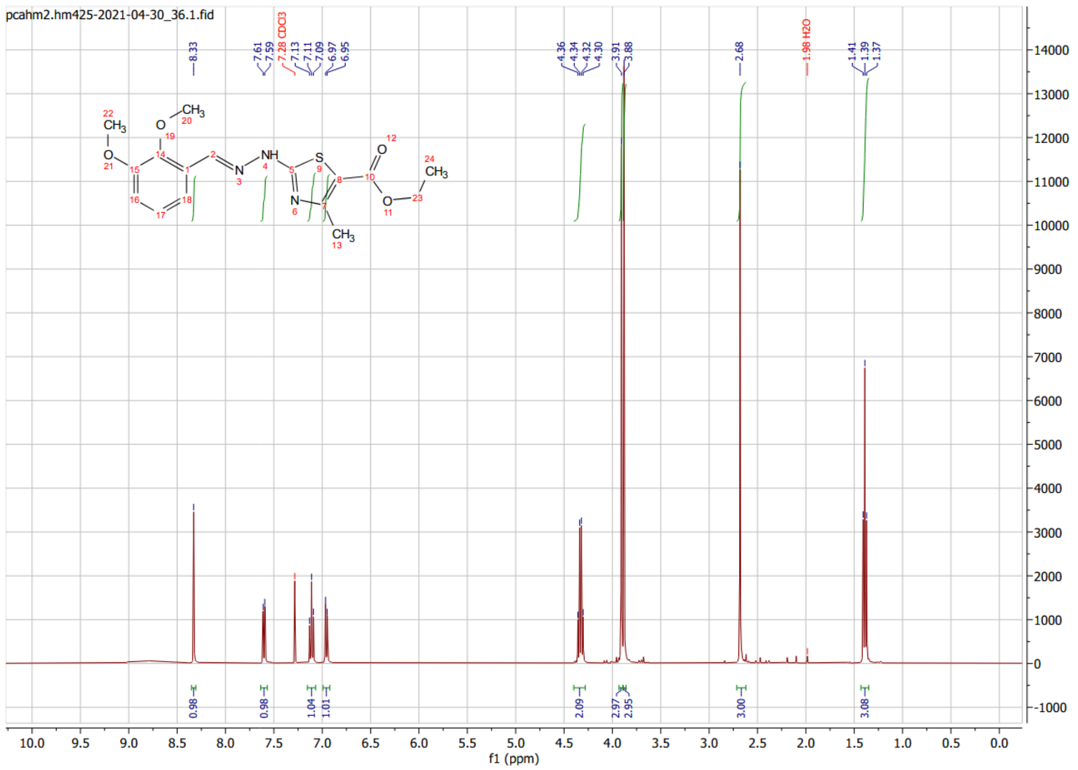

Figure S14. ^{1}H NMR Spectrum of **3g**

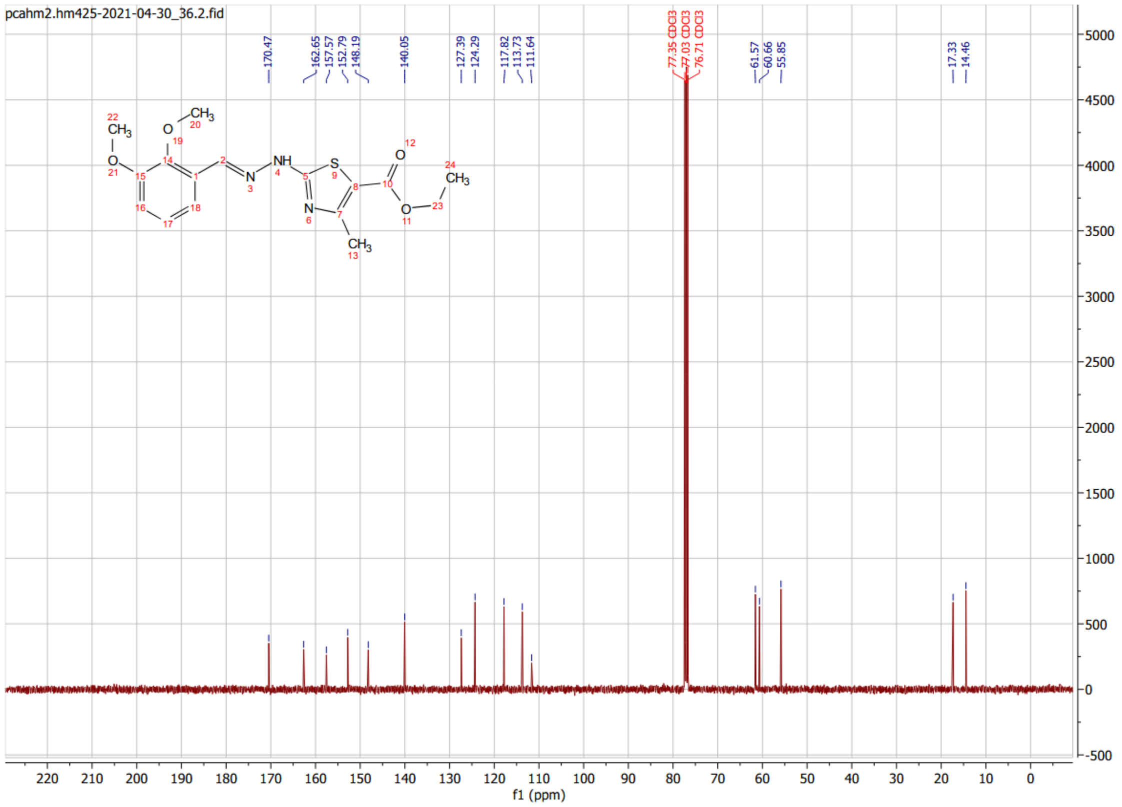

Figure S15. ^{13}C NMR Spectrum of **3g**

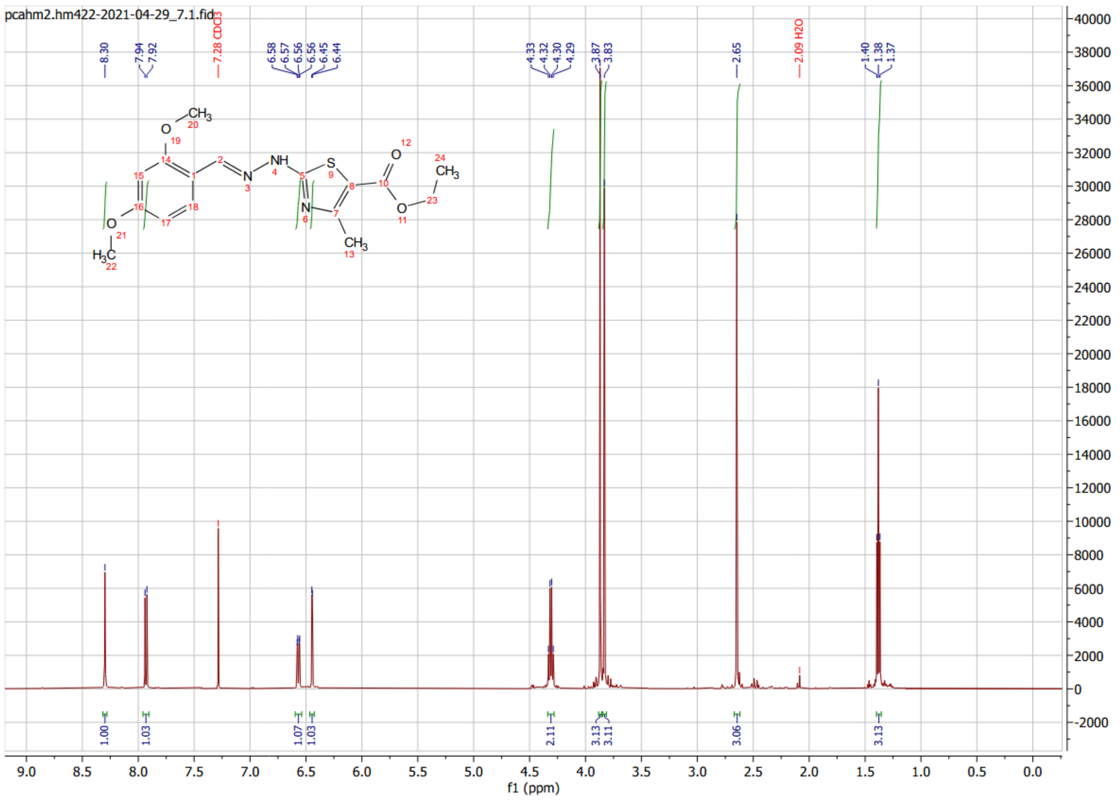

Figure S16.^{1}H NMR Spectrum of **3h**

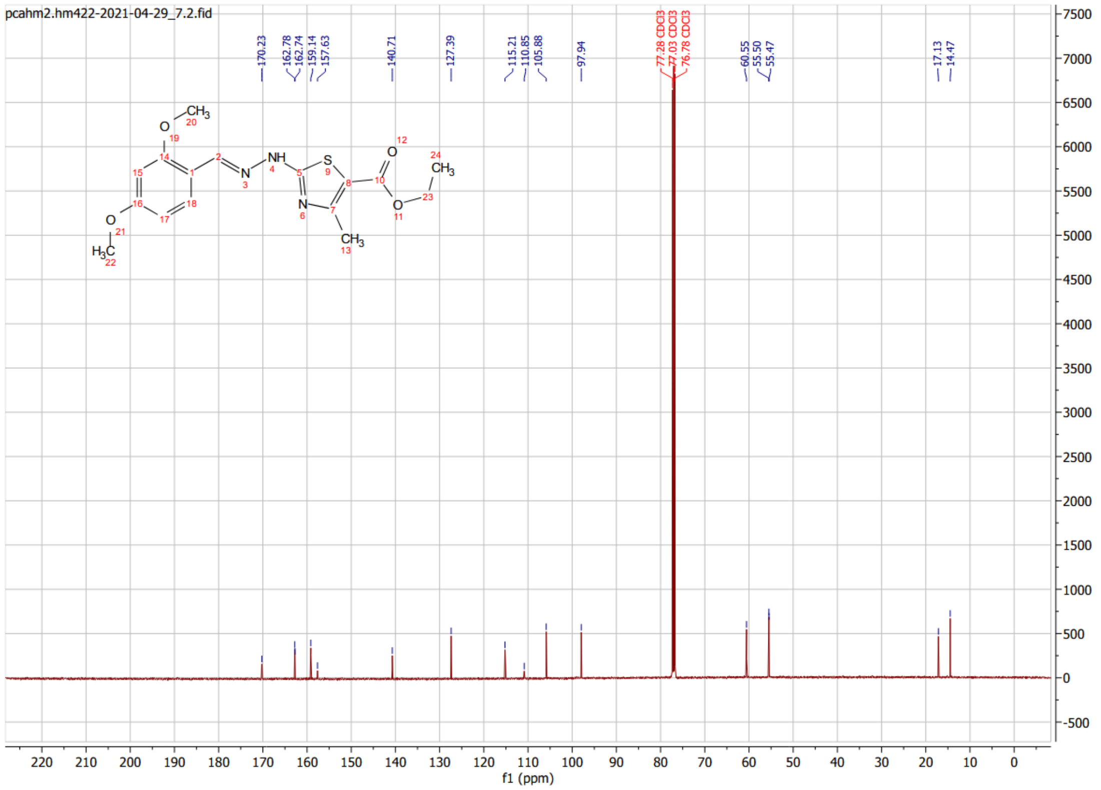

Figure S17.^{13}C NMR Spectrum of **3h**

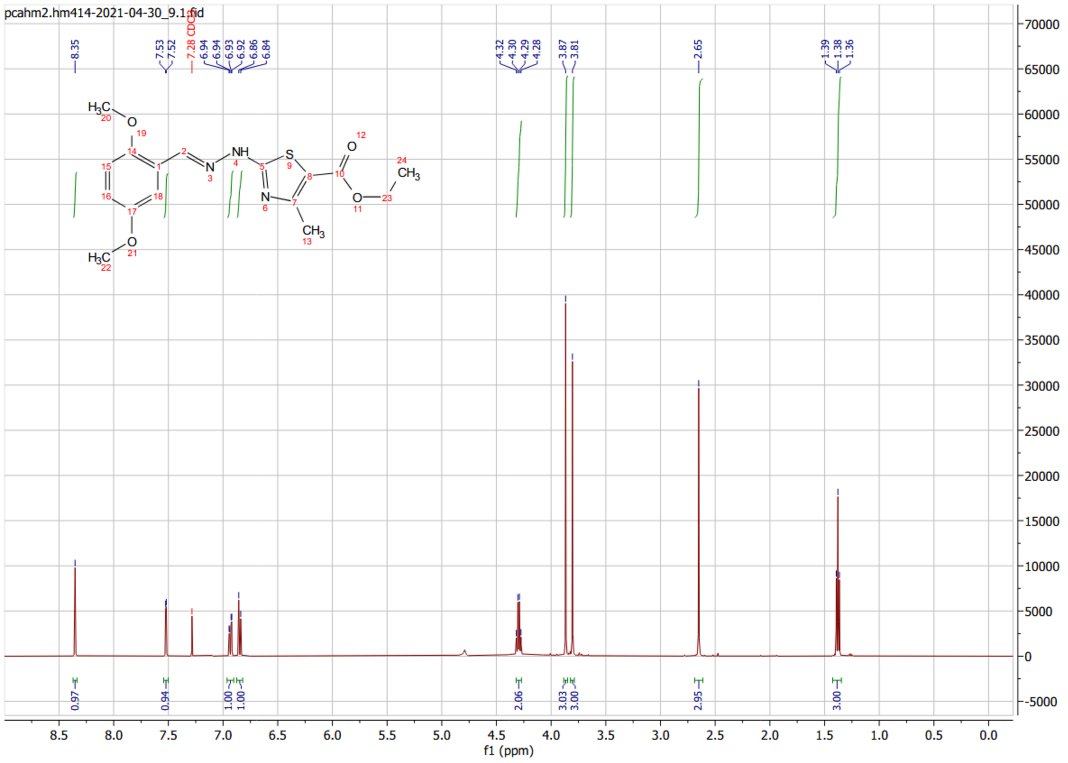

Figure S18.^{1}H NMR Spectrum of **3i**

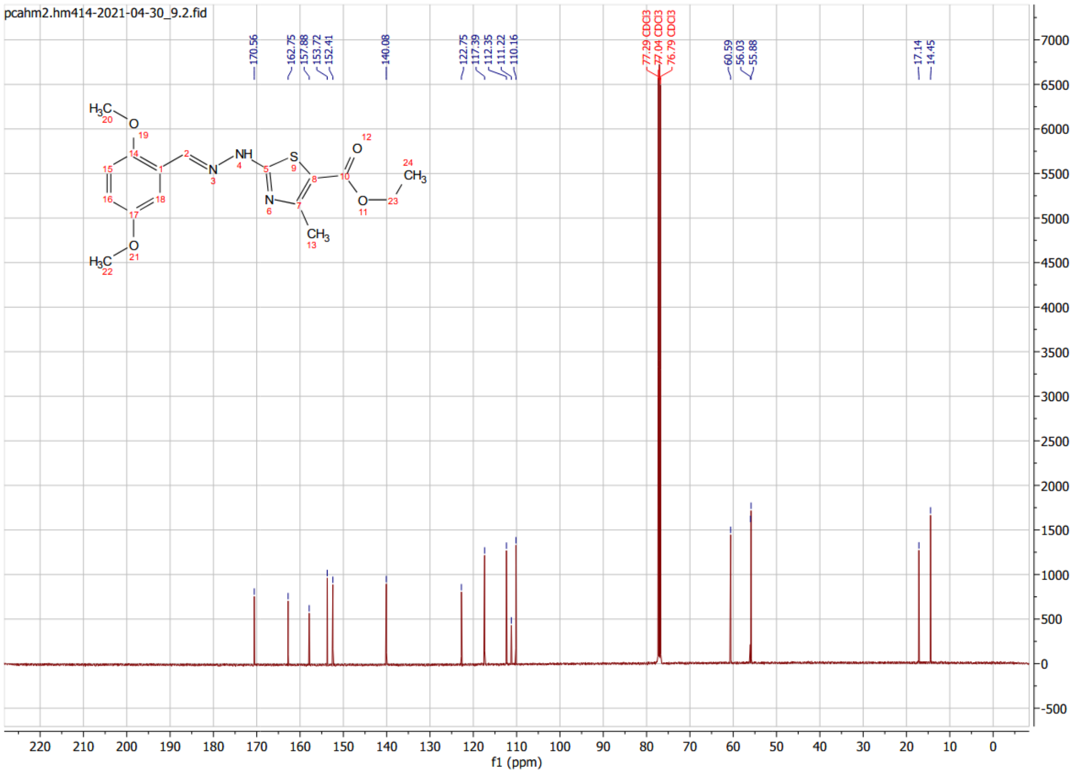

Figure S19.^{13}C NMR Spectrum of **3i**

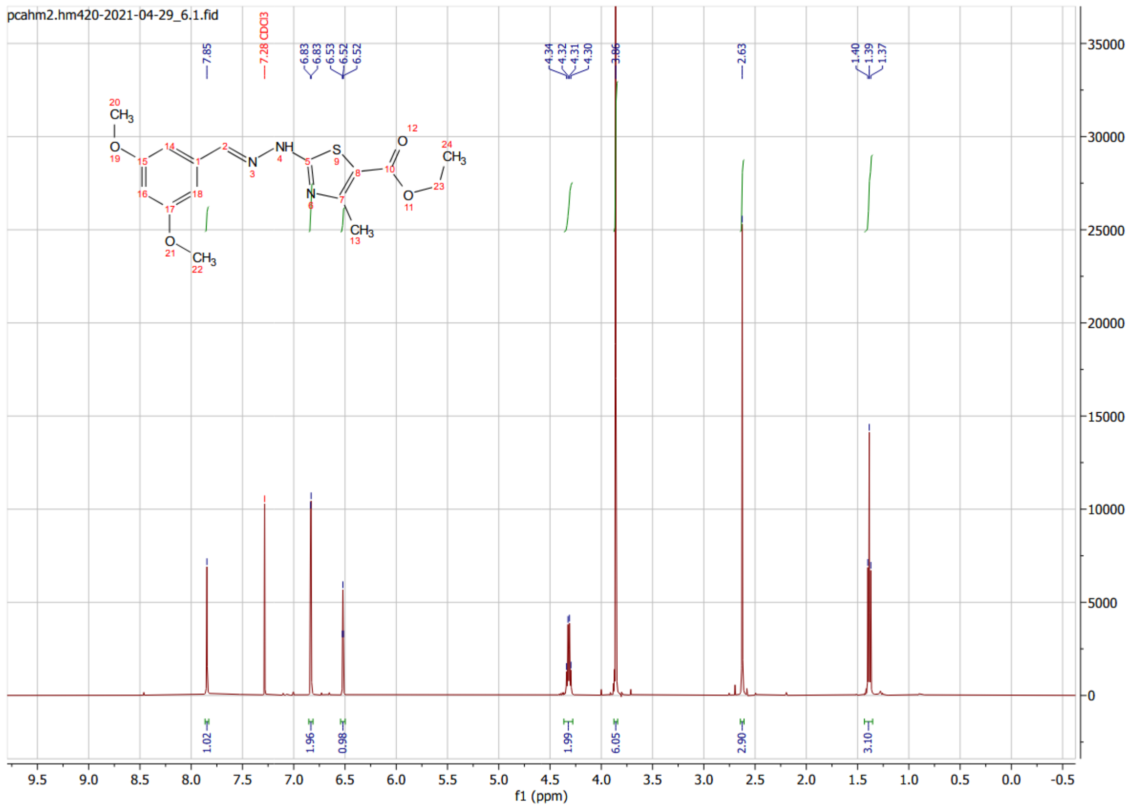

Figure S20.^{1}H NMR Spectrum of **3j**

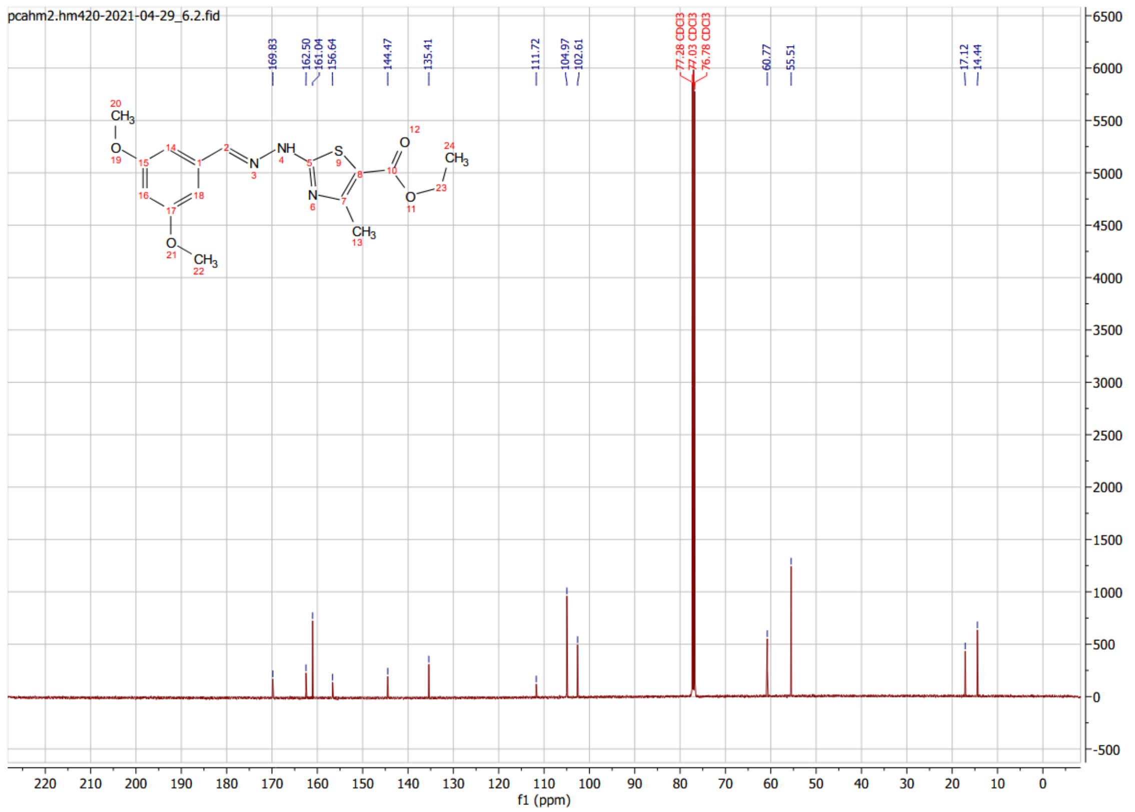

Figure S21. ^{13}C NMR Spectrum of **3j**

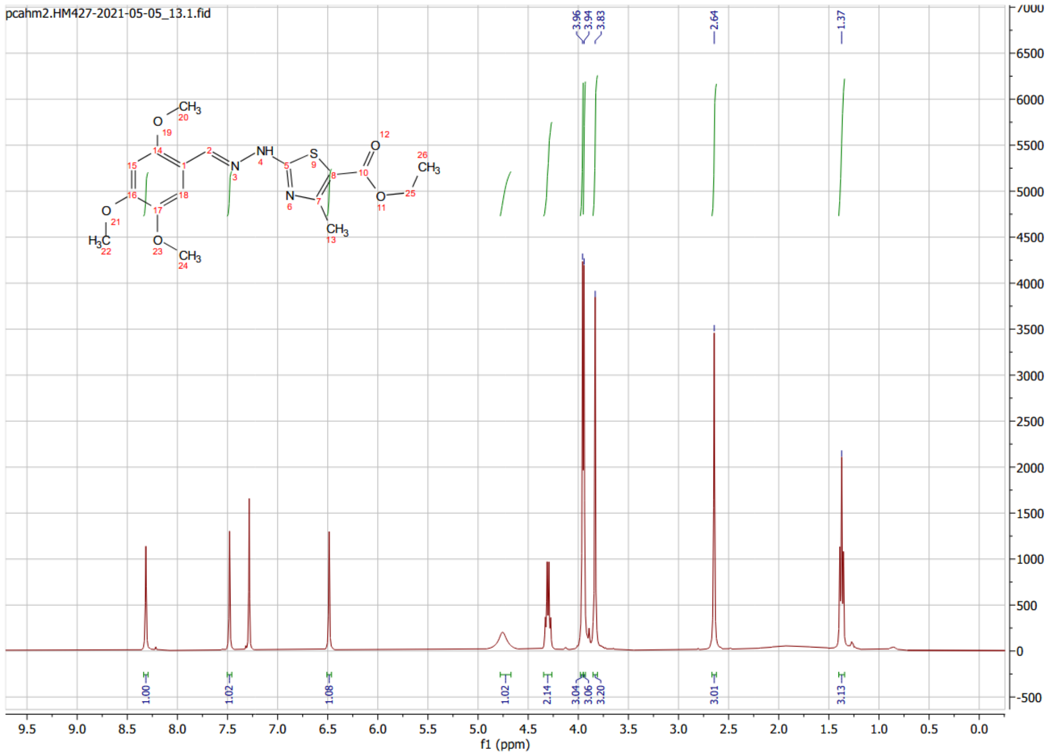

Figure S22. ^{1}H NMR Spectrum of **3k**

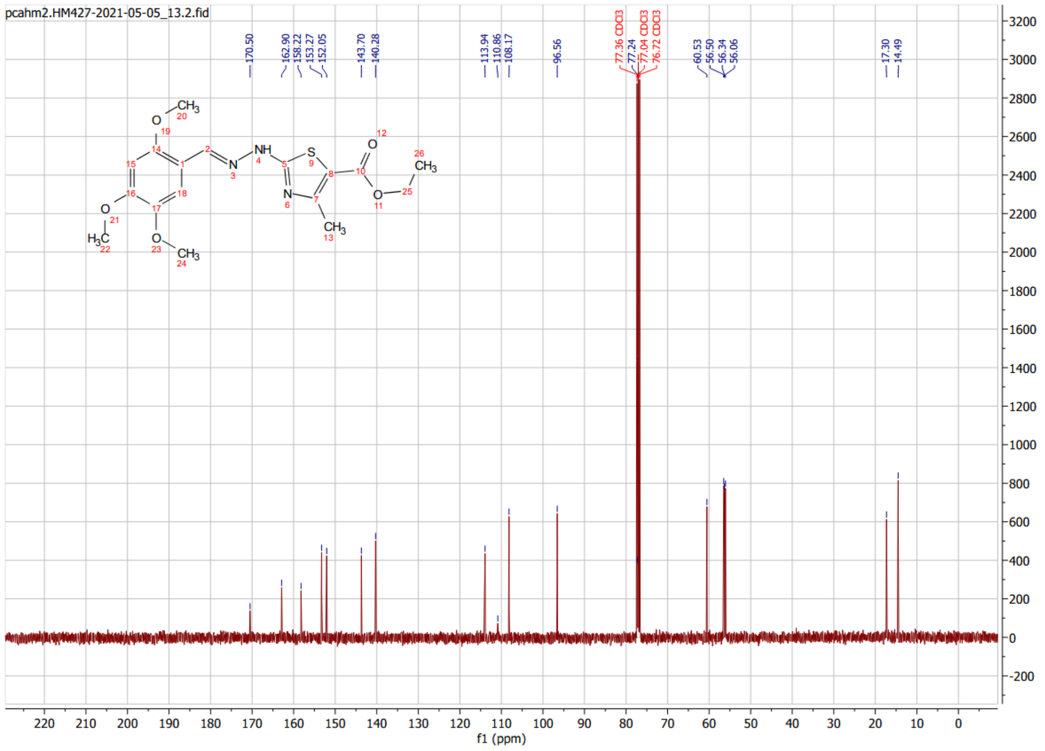

Figure S23. ^{13}C NMR Spectrum of **3k**

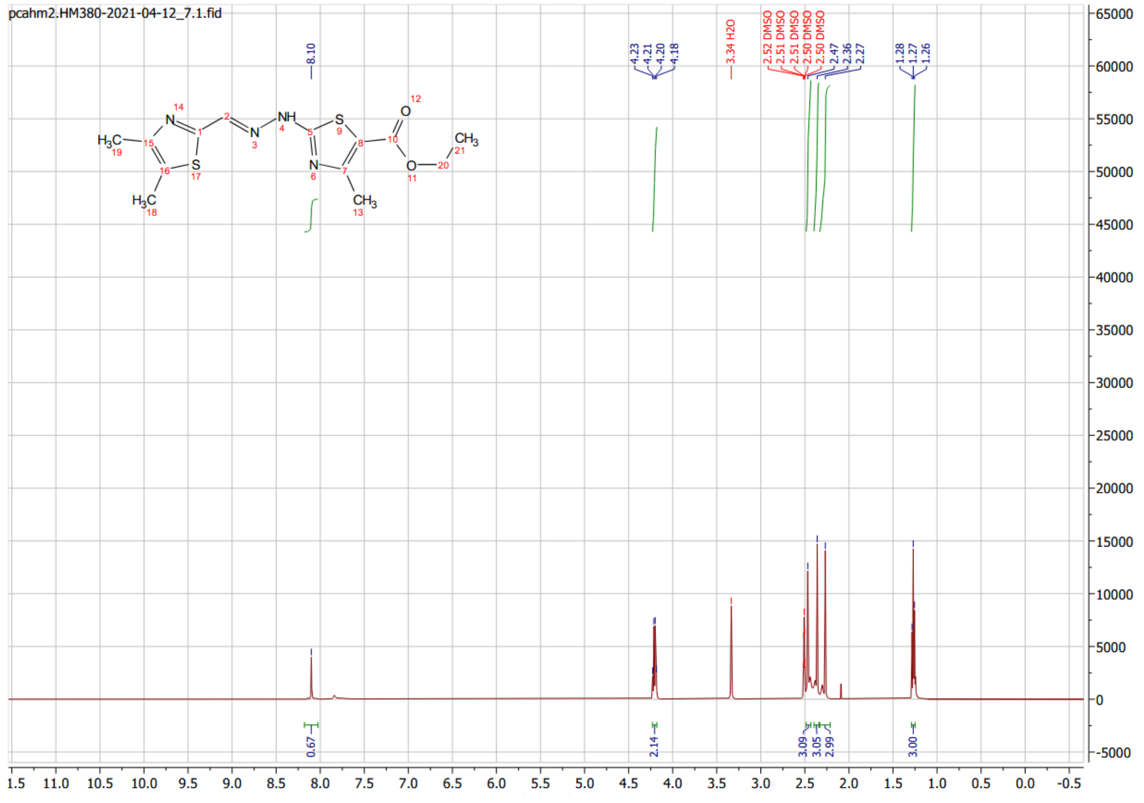

Figure S24. ^{1}H NMR Spectrum of **3l**

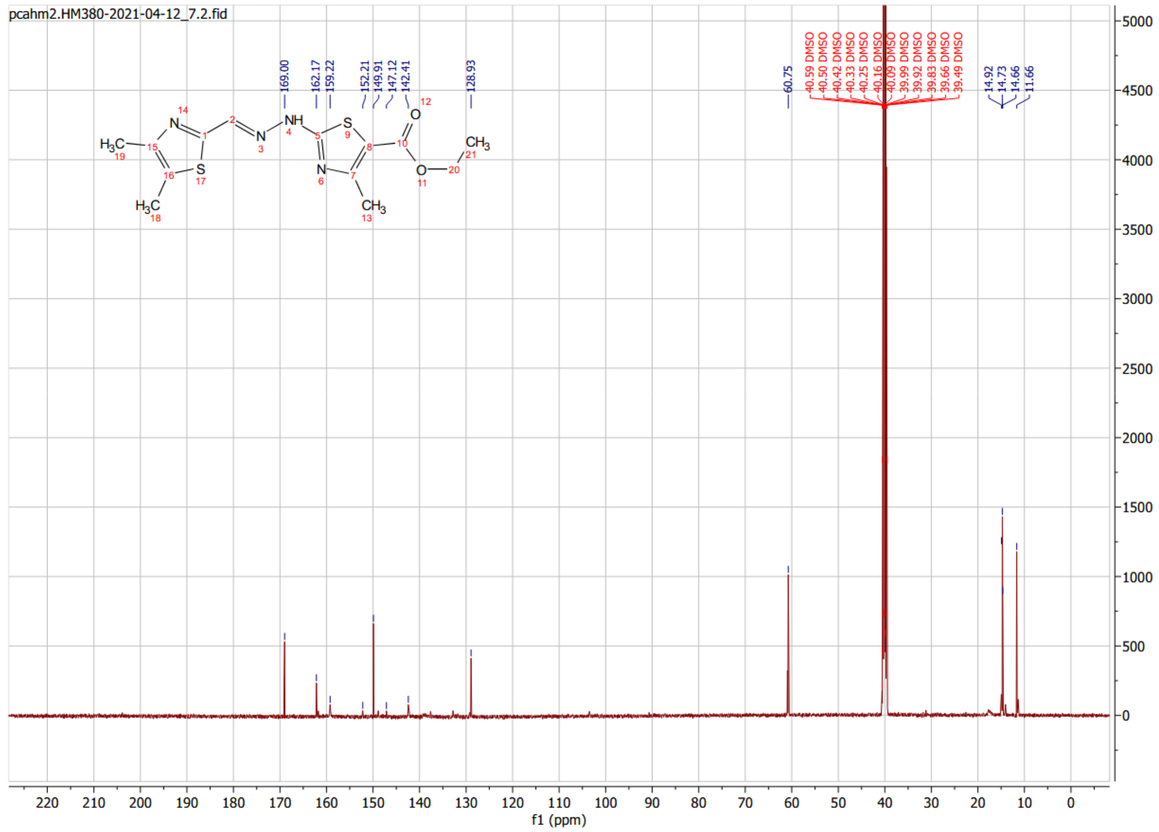

Figure S25. ^{13}C NMR Spectrum of **3l**

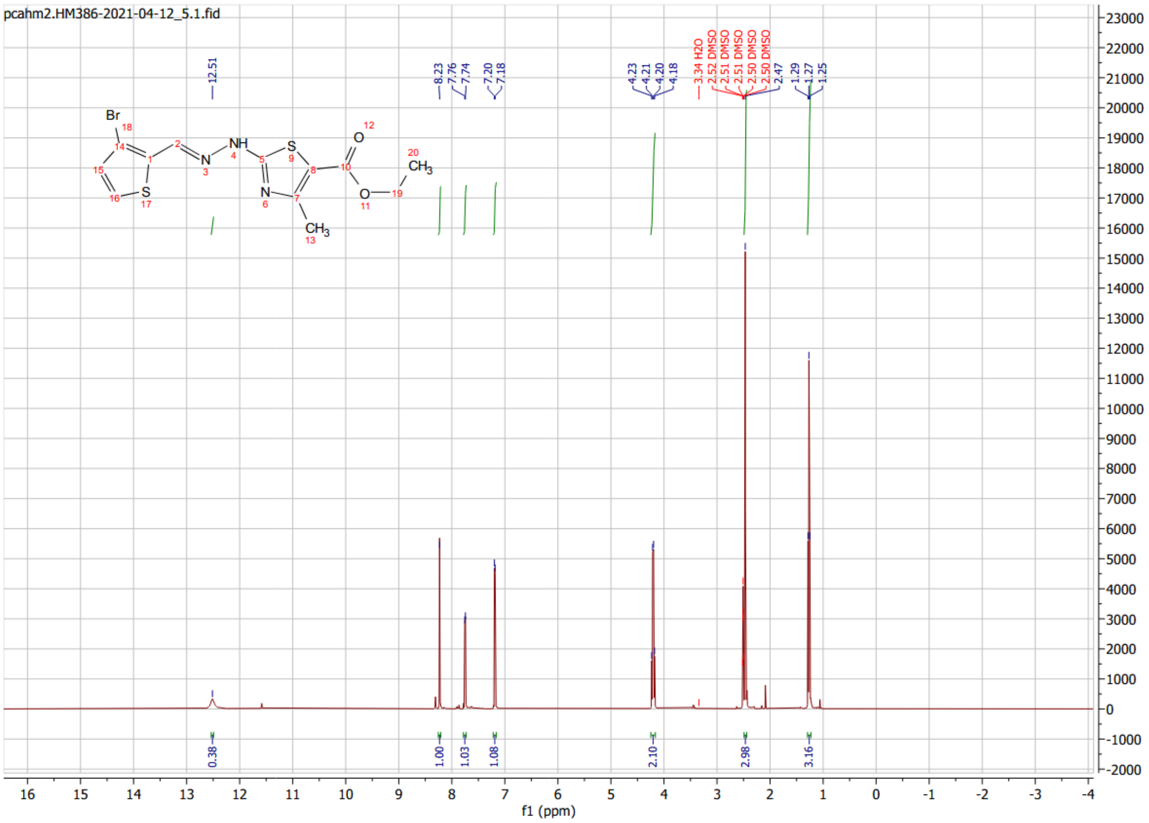

Figure S26. ^{1}H NMR Spectrum of **3m**

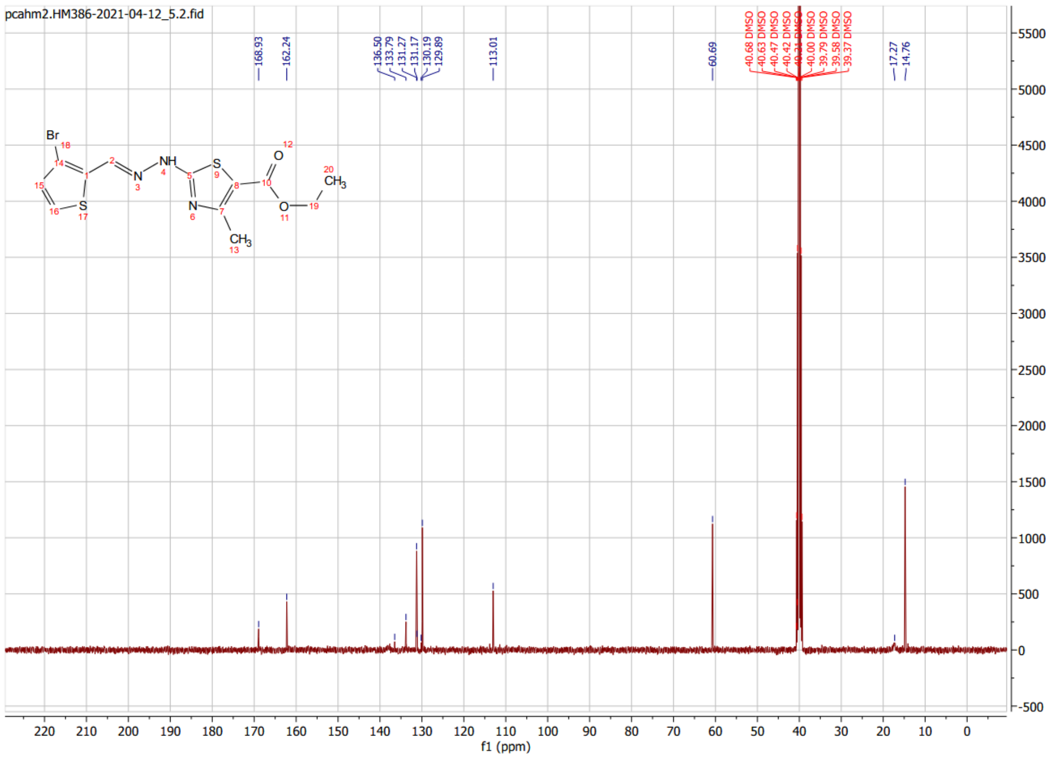

Figure S27. ^{13}C NMR Spectrum of **3m**

Mass Spectrum of compounds 3a-m

Mass Spectrometry
Analytical Services
School of Chemistry

The University of Nottingham

Sample-ID	h_meh_HM395	Lab	C13
Submitter	Hasnain Mehmood	Supervisor	Simon Woodward
Analysis Name	h_meh_HM395_615293_39_01_31981.d	Acquisition Date	5/5/2021 4:23:52 PM
Ionisation Mode	ESI Positive	Instrument	Bruker MicroTOF

+MS, 0.7-0.9min #42-53

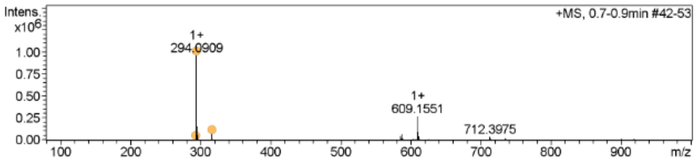

#	m/z	I %
1	294.0909	100.0
2	295.0935	16.4
3	296.0895	6.0
4	316.0723	6.8
5	317.0751	1.1
6	585.1573	4.4
7	586.1601	1.4
8	587.1718	6.8
9	588.1748	2.2
10	589.1722	1.0
11	603.1681	1.5
12	609.1551	28.1
13	610.1583	9.4
14	611.1554	4.2
15	612.1581	1.1
16	712.3975	3.8
17	713.4002	1.7
18	734.3793	2.0
19	900.2216	1.5
20	918.2324	1.3

Generate Molecular Formula Parameters

Charge	Tolerance	sigma limit	H/C Ratio	Electron Conf.	Nitrogen Rule	Chrom.BackGround	Calibration
+1	6 ppm	0.08	3 - 0	both	false	false	TRUE

Expected Formula C13 H15 N3 O3 S1 **Adduct(s):** H, Na, NH4, C3H5N2, radical

#	meas. m/z	theo. m/z	|Err|[ppm]	Sigma	Formula	Adduct	Adduct Mass
1	294.0909	294.0907	0.70	0.0018	C13H16N3O3S	M+H	1.0078
1	316.0723	316.0726	1.10	0.0017	C13H15N3NaO3S	M+Na	22.9898

Note: Sigma fits < 0.05 indicates high probability of correct MF

Figure S28. Mass Spectrum of **3a**

Mass Spectrometry
Analytical Services
School of Chemistry

The University of Nottingham

Sample-ID	h_meh_hm418	Lab	C13
Submitter	Hasnain Mehmood	Supervisor	Simon Woodward
Analysis Name	h_meh_hm418_615365_65_01_32013.d	Acquisition Date	5/6/2021 12:10:07 PM
Ionisation Mode	ESI Positive	Instrument	Bruker MicroTOF

+MS, 0.7-0.9min #42-53

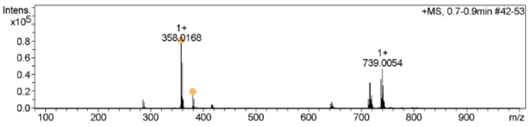

#	m/z	I %
1	285.9967	13.2
2	358.0168	100.0
3	359.0198	18.3
4	360.0146	70.2
5	361.0170	11.9
6	362.0117	14.3
7	379.9994	20.5
8	381.9964	14.3
9	713.0111	15.3
10	715.0173	40.1
11	716.0206	13.6
12	717.0191	39.9
13	718.0212	13.6
14	719.0183	20.1
15	737.0077	45.3
16	738.0119	15.8
17	739.0054	61.2
18	740.0081	21.3
19	741.0032	35.5
20	742.0049	12.0

Generate Molecular Formula Parameters

Charge	Tolerance	sigma limit	H/C Ratio	Electron Conf.	Nitrogen Rule	Chrom.BackGround	Calibration
+1	6 ppm	0.08	3 - 0	both	false	false	TRUE

Expected Formula C14 H13 Cl2 N3 O2 S1 **Adduct(s):** H, Na, NH4, C3H5N2, radical

#	meas. m/z	theo. m/z	|Err|[ppm]	Sigma	Formula	Adduct	Adduct Mass
1	358.0168	358.0178	2.90	0.0041	C14H14Cl2N3O2S	M+H	1.0078
1	379.9994	379.9998	1.10	0.0058	C14H13Cl2N3NaO2S	M+Na	22.9898

Note: Sigma fits < 0.05 indicates high probability of correct MF

Figure S29. Mass Spectrum of **3b**

Mass Spectrometry
Analytical Services
School of Chemistry

The University of Nottingham

Sample-ID	h_meh_HM406	Lab	C13
Submitter	Hasnain Mehmood	Supervisor	Simon Woodward
Analysis Name	h_meh_HM406_615295_41_01_31983.d	Acquisition Date	5/5/2021 4:29:07 PM
Ionisation Mode	ESI Positive	Instrument	Bruker MicroTOF

+MS, 0.7-0.9min #41-52

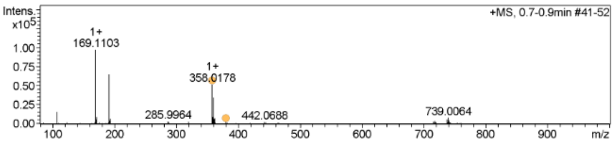

#	m/z	I %
1	107.0406	16.4
2	169.1103	100.0
3	170.1122	5.9
4	171.1068	9.2
5	191.0922	66.8
6	192.0944	4.1
7	193.0883	6.9
8	285.9964	3.1
9	320.1063	2.7
10	358.0178	52.9
11	359.0207	9.2
12	360.0148	35.7
13	361.0175	6.4
14	362.0123	7.4
15	715.0250	2.7
16	717.0254	3.7
17	737.0087	5.9
18	739.0064	8.4
19	740.0082	2.8
20	741.0037	4.6

Generate Molecular Formula Parameters

Charge	Tolerance	sigma limit	H/C Ratio	Electron Conf.	Nitrogen Rule	Chrom.BackGround	Calibration
+1	6 ppm	0.08	3 - 0	both	false	false	TRUE

Expected Formula C14 H13 Cl2 N3 O2 S1 **Adduct(s):** H, Na, NH4, C3H5N2, radical

#	meas. m/z	theo. m/z	|Err|[ppm]	Sigma	Formula	Adduct	Adduct Mass
1	358.0178	358.0178	0.00	0.0115	C14H14Cl2N3O2S	M+H	1.0078
1	379.9995	379.9998	0.70	0.0254	C14H13Cl2N3NaO2S	M+Na	22.9898

Note: Sigma fits < 0.05 indicates high probability of correct MF

Figure S30. Mass Spectrum of **3c**

Mass Spectrometry
Analytical Services
School of Chemistry

The University of Nottingham

Sample-ID	h_meh_HM410	Lab	C13
Submitter	Hasnain Mehmood	Supervisor	Simon Woodward
Analysis Name	h_meh_HM410_615090_86_01_31898.d	Acquisition Date	4/30/2021 4:02:20 PM
Ionisation Mode	ESI Positive	Instrument	Bruker MicroTOF

+MS, 0.7-0.9min #42-53

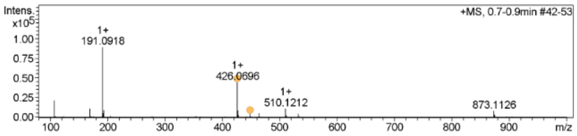

#	m/z	I %
1	107.0389	24.2
2	169.1096	12.4
3	175.0263	1.5
4	190.0859	2.0
5	191.0918	100.0
6	192.0938	6.1
7	193.0880	10.2
8	205.0381	2.0
9	354.0490	1.8
10	426.0696	50.1
11	427.0731	9.8
12	428.0679	3.3
13	448.0506	4.9
14	464.0445	5.9
15	510.1212	12.5
16	511.1234	2.9
17	532.1012	4.9
18	873.1126	9.3
19	874.1162	3.5
20	875.1116	1.7

Generate Molecular Formula Parameters

Charge	Tolerance	sigma limit	H/C Ratio	Electron Conf.	Nitrogen Rule	Chrom.BackGround	Calibration
+1	6 ppm	0.08	3 - 0	both	false	false	TRUE

Expected Formula C16 H13 F6 N3 O2 S1 **Adduct(s):** H, Na, NH4, C3H5N2, radical

#	meas. m/z	theo. m/z	|Err|[ppm]	Sigma	Formula	Adduct	Adduct Mass
1	426.0696	426.0705	2.20	0.0012	C16H14F6N3O2S	M+H	1.0078
1	448.0506	448.0525	4.30	0.0345	C16H13F6N3NaO2S	M+Na	22.9898

Note: Sigma fits < 0.05 indicates high probability of correct MF

Figure S31. Mass Spectrum of **3d**

Mass Spectrometry
Analytical Services
School of Chemistry

The University of Nottingham

Sample-ID	h_meh_HM398	Lab	C13
Submitter	Hasnain Mehmood	Supervisor	Simon Woodward
Analysis Name	h_meh_HM398_615294_40_01_31982.d	Acquisition Date	5/5/2021 4:26:24 PM
Ionisation Mode	ESI Positive	Instrument	Bruker MicroTOF

+MS, 0.7-0.9min #41-52

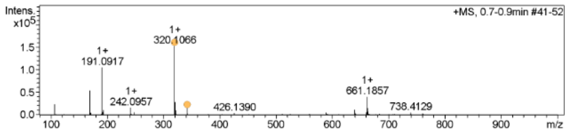

#	m/z	I %
1	107.0375	15.4
2	169.1093	35.8
3	171.1057	3.3
4	191.0917	69.1
5	192.0935	4.1
6	193.0880	6.8
7	242.0957	10.3
8	248.0849	3.5
9	320.1066	100.0
10	321.1095	18.6
11	322.1053	6.4
12	342.0882	9.8
13	589.1621	3.2
14	639.2036	7.3
15	640.2061	2.6
16	660.4029	3.5
17	661.1857	26.4
18	662.1888	9.5
19	663.1848	4.4
20	738.4129	3.3

Generate Molecular Formula Parameters

Charge	Tolerance	sigma limit	H/C Ratio	Electron Conf.	Nitrogen Rule	Chrom.BackGround	Calibration
+1	6 ppm	0.08	3 - 0	both	false	false	TRUE

Expected Formula C15 H17 N3 O3 S1 **Adduct(s):** H, Na, NH4, C3H5N2, radical

#	meas. m/z	theo. m/z	|Err|[ppm]	Sigma	Formula	Adduct	Adduct Mass
1	320.1066	320.1063	0.70	0.0016	C15H18N3O3S	M+H	1.0078
1	342.0882	342.0883	0.30	0.0030	C15H17N3NaO3S	M+Na	22.9898

Note: Sigma fits < 0.05 indicates high probability of correct MF

Figure S32. Mass Spectrum of **3e**

Mass Spectrometry
Analytical Services
School of Chemistry

The University of Nottingham

Sample-ID	h_meh_hm416	Lab	C13
Submitter	Hasnain Mehmood	Supervisor	Simon Woodward
Analysis Name	h_meh_hm416_615364_64_01_32012.d	Acquisition Date	5/6/2021 12:07:38 PM
Ionisation Mode	ESI Positive	Instrument	Bruker MicroTOF

+MS, 0.7-0.9min #42-53

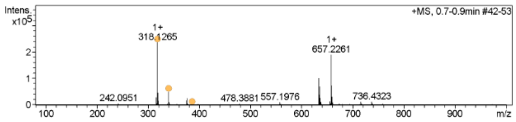

#	m/z	I %
1	316.1110	12.1
2	318.1265	100.0
3	319.1294	19.5
4	320.1259	6.2
5	340.1080	21.2
6	341.1110	4.2
7	375.1840	6.7
8	376.1356	10.5
9	557.1976	4.1
10	633.2287	42.8
11	634.2320	16.7
12	635.2411	29.7
13	636.2443	10.7
14	637.2434	4.4
15	655.2106	5.5
16	657.2261	80.4
17	658.2290	31.2
18	659.2270	13.0
19	715.2200	5.1
20	736.4323	5.2

Generate Molecular Formula Parameters

Charge	Tolerance	sigma limit	H/C Ratio	Electron Conf.	Nitrogen Rule	Chrom.BackGround	Calibration
+1	6 ppm	0.08	3 - 0	both	false	false	TRUE

Expected Formula C16 H19 N3 O2 S1 **Adduct(s):** H, Na, NH4, C3H5N2, radical

#	meas. m/z	theo. m/z	|Err|[ppm]	Sigma	Formula	Adduct	Adduct Mass
1	318.1265	318.1271	1.90	0.0025	C16H20N3O2S	M+H	1.0078
1	340.1080	340.1090	3.10	0.0019	C16H19N3NaO2S	M+Na	22.9898

Note: Sigma fits < 0.05 indicates high probability of correct MF

Figure S33. Mass Spectrum of **3f**

Mass Spectrometry
Analytical Services
School of Chemistry

The University of Nottingham

Sample-ID	h_meh_hm425	Lab	C13
Submitter	Hasnain Mehmood	Supervisor	Simon Woodward
Analysis Name	h_meh_hm425_615366_66_01_32014.d	Acquisition Date	5/6/2021 12:12:37 PM
Ionisation Mode	ESI Positive	Instrument	Bruker MicroTOF

+MS, 0.7-0.9min #42-53

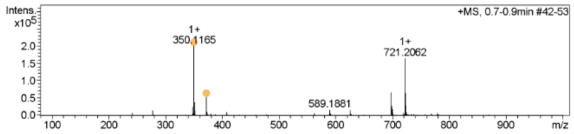

#	m/z	I %
1	242.0956	3.7
2	278.0951	6.6
3	348.1010	11.8
4	350.1165	100.0
5	351.1192	19.3
6	352.1154	6.8
7	372.0980	26.5
8	373.1013	5.0
9	407.1736	3.9
10	408.1278	4.3
11	589.1881	7.7
12	625.1889	7.1
13	697.2090	33.2
14	698.2115	13.1
15	699.2143	8.5
16	719.1931	3.5
17	721.2062	82.2
18	722.2086	32.6
19	723.2077	14.3
20	724.2082	4.2

Generate Molecular Formula Parameters

Charge	Tolerance	sigma limit	H/C Ratio	Electron Conf.	Nitrogen Rule	Chrom.BackGround	Calibration
+1	6 ppm	0.08	3 - 0	both	false	false	TRUE

Expected Formula C16 H19 N3 O4 S1 **Adduct(s):** H, Na, NH4, C3H5N2, radical

#	meas. m/z	theo. m/z	|Err|[ppm]	Sigma	Formula	Adduct	Adduct Mass
1	350.1165	350.1169	1.20	0.0024	C16H20N3O4S	M+H	1.0078
1	372.0980	372.0988	2.30	0.0036	C16H19N3NaO4S	M+Na	22.9898

Note: Sigma fits < 0.05 indicates high probability of correct MF

Figure S34. Mass Spectrum of **3g**

Mass Spectrometry
Analytical Services
School of Chemistry

The University of Nottingham

Sample-ID	h_meh_HM422	Lab	C13
Submitter	Hasnain Mehmood	Supervisor	Simon Woodward
Analysis Name	h_meh_HM422_615088_84_01_31896.d	Acquisition Date	4/30/2021 3:57:22 PM
Ionisation Mode	ESI Positive	Instrument	Bruker MicroTOF

+MS, 0.7-0.9min #42-53

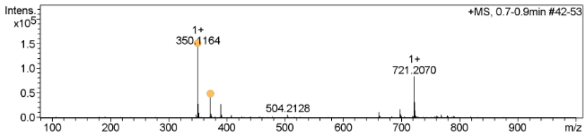

#	m/z	I %
1	348.1013	3.9
2	350.1164	100.0
3	351.1192	20.1
4	352.1155	6.7
5	372.0982	28.6
6	373.1019	5.7
7	390.1479	19.5
8	391.1507	4.3
9	408.1270	3.6
10	504.2128	4.0
11	661.2097	7.7
12	697.2103	12.1
13	698.2123	4.9
14	719.1938	2.9
15	721.2070	58.7
16	722.2098	22.4
17	723.2079	10.2
18	724.2107	3.0
19	768.4231	3.9
20	779.2038	3.1

Generate Molecular Formula Parameters

Charge	Tolerance	sigma limit	H/C Ratio	Electron Conf.	Nitrogen Rule	Chrom.BackGround	Calibration
+1	6 ppm	0.08	3 - 0	both	false	false	TRUE

Expected Formula C16 H19 N3 O4 S1 **Adduct(s):** H, Na, NH4, C3H5N2, radical

#	meas. m/z	theo. m/z	|Err|[ppm]	Sigma	Formula	Adduct	Adduct Mass
1	350.1164	350.1169	1.50	0.0034	C16H20N3O4S	M+H	1.0078
1	372.0982	372.0988	1.70	0.0021	C16H19N3NaO4S	M+Na	22.9898

Note: Sigma fits < 0.05 indicates high probability of correct MF

Figure S35. Mass Spectrum of **3h**

Mass Spectrometry
Analytical Services
School of Chemistry

The University of Nottingham

Sample-ID	h_meh_hm414	Lab	C13
Submitter	Hasnain Mehmood	Supervisor	Simon Woodward
Analysis Name	h_meh_hm414_615363_63_01_32011.d	Acquisition Date	5/6/2021 12:05:11 PM
Ionisation Mode	ESI Positive	Instrument	Bruker MicroTOF

+MS, 0.7-0.9min #42-53

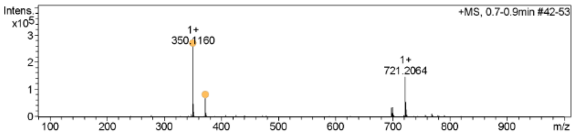

#	m/z	I %
1	348.1007	3.4
2	350.1160	100.0
3	351.1186	18.8
4	352.1153	6.9
5	372.0975	26.6
6	373.1009	5.3
7	408.1280	2.3
8	426.1471	2.1
9	697.2086	12.6
10	698.2115	5.0
11	699.2217	13.2
12	700.2251	4.9
13	701.2223	2.1
14	721.2064	57.2
15	722.2082	21.6
16	723.2068	10.0
17	724.2079	2.7
18	757.2291	2.3
19	768.4227	4.0
20	790.4034	2.0

Generate Molecular Formula Parameters

Charge	Tolerance	sigma limit	H/C Ratio	Electron Conf.	Nitrogen Rule	Chrom.BackGround	Calibration
+1	6 ppm	0.08	3 - 0	both	false	false	TRUE

Expected Formula C16 H19 N3 O4 S1 **Adduct(s):** H, Na, NH4, C3H5N2, radical

#	meas. m/z	theo. m/z	|Err|[ppm]	Sigma	Formula	Adduct	Adduct Mass
1	350.1160	350.1169	2.60	0.0039	C16H20N3O4S	M+H	1.0078
1	372.0975	372.0988	3.60	0.0021	C16H19N3NaO4S	M+Na	22.9898

Note: Sigma fits < 0.05 indicates high probability of correct MF

Figure S36. Mass Spectrum of **3i**

Mass Spectrometry
Analytical Services
School of Chemistry

The University of Nottingham

Sample-ID	h_meh_HM420	Lab	C13
Submitter	Hasnain Mehmood	Supervisor	Simon Woodward
Analysis Name	h_meh_HM420_615087_83_01_31895.d	Acquisition Date	4/30/2021 3:54:52 PM
Ionisation Mode	ESI Positive	Instrument	Bruker MicroTOF

+MS, 0.7-0.9min #42-53

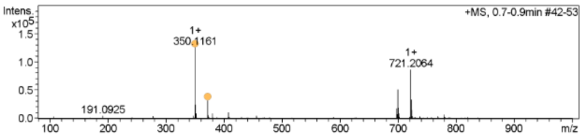

#	m/z	I %
1	191.0925	3.8
2	348.1009	3.7
3	350.1161	100.0
4	351.1191	19.7
5	352.1154	6.9
6	372.0977	25.5
7	373.1010	4.7
8	380.1263	6.7
9	408.1220	8.8
10	456.1489	3.5
11	697.2089	14.1
12	698.2114	5.8
13	699.2238	41.3
14	700.2266	15.5
15	701.2251	7.0
16	721.2064	68.8
17	722.2086	26.7
18	723.2069	11.9
19	724.2081	3.3
20	779.2053	5.4

Generate Molecular Formula Parameters

Charge	Tolerance	sigma limit	H/C Ratio	Electron Conf.	Nitrogen Rule	Chrom.BackGround	Calibration
+1	6 ppm	0.08	3 - 0	both	false	false	TRUE

Expected Formula C16 H19 N3 O4 S1 **Adduct(s):** H, Na, NH4, C3H5N2, radical

#	meas. m/z	theo. m/z	|Err|[ppm]	Sigma	Formula	Adduct	Adduct Mass
1	350.1161	350.1169	2.40	0.0013	C16H20N3O4S	M+H	1.0078
1	372.0977	372.0988	3.00	0.0054	C16H19N3NaO4S	M+Na	22.9898

Note: Sigma fits < 0.05 indicates high probability of correct MF

Figure S37. Mass Spectrum of **3j**

Mass Spectrometry
Analytical Services
School of Chemistry

The University of Nottingham

Sample-ID	h_meh_HM427	Lab	C13
Submitter	Hasnain Mehmood	Supervisor	Simon Woodward
Analysis Name	h_meh_HM427_615296_42_01_31984.d	Acquisition Date	5/5/2021 4:31:36 PM
Ionisation Mode	ESI Positive	Instrument	Bruker MicroTOF

+MS, 0.7-0.9min #42-53

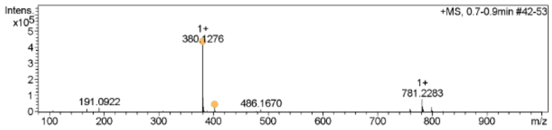

#	m/z	I %
1	107.0401	1.9
2	169.1102	4.1
3	191.0922	6.0
4	308.1067	1.2
5	380.1276	100.0
6	381.1300	21.2
7	382.1271	7.3
8	383.1281	1.3
9	389.1703	1.8
10	402.1093	6.1
11	403.1119	1.2
12	486.1670	3.6
13	759.2463	4.1
14	760.2479	1.8
15	781.2283	19.4
16	782.2310	7.8
17	783.2281	3.6
18	798.4346	6.9
19	799.4369	3.4
20	800.4373	1.2

Generate Molecular Formula Parameters

Charge	Tolerance	sigma limit	H/C Ratio	Electron Conf.	Nitrogen Rule	Chrom.BackGround	Calibration
+1	6 ppm	0.08	3 - 0	both	false	false	TRUE

Expected Formula C17 H21 N3 O5 S1 **Adduct(s):** H, Na, NH4, C3H5N2, radical

#	meas. m/z	theo. m/z	|Err|[ppm]	Sigma	Formula	Adduct	Adduct Mass
1	380.1276	380.1275	0.30	0.0028	C17H22N3O5S	M+H	1.0078
1	402.1093	402.1094	0.20	0.0029	C17H21N3NaO5S	M+Na	22.9898

Note: Sigma fits < 0.05 indicates high probability of correct MF

Figure S38. Mass Spectrum of **3k**

Mass Spectrometry
Analytical Services
School of Chemistry

The University of Nottingham

Sample-ID	h_meh_hm380	Lab	C13
Submitter	Hasnain Mehmood	Supervisor	Simon Woodward
Analysis Name	h_meh_hm380_613469_68_01_31275.d	Acquisition Date	4/12/2021 4:08:11 PM
Ionisation Mode	ESI Positive	Instrument	Bruker MicroTOF

+MS, 0.7-0.9min #41-52

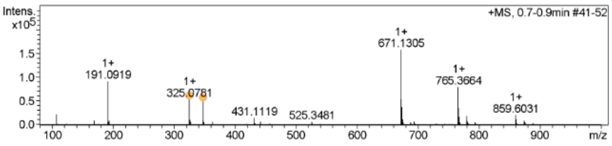

#	m/z	I %
1	107.0396	13.9
2	169.1098	5.3
3	191.0919	58.2
4	193.0882	5.5
5	325.0781	33.7
6	326.0811	6.3
7	347.0604	31.2
8	431.1119	8.9
9	671.1305	100.0
10	672.1329	34.1
11	673.1293	23.9
12	674.1303	6.8
13	765.3664	50.3
14	766.3701	22.8
15	767.3683	10.3
16	779.3821	11.5
17	780.3850	5.5
18	859.6031	12.7
19	860.6059	6.9
20	873.6177	5.4

Generate Molecular Formula Parameters

Charge	Tolerance	sigma limit	H/C Ratio	Electron Conf.	Nitrogen Rule	Chrom.BackGround	Calibration
+1	6 ppm	0.08	3 - 0	both	false	false	TRUE

Expected Formula C13 H16 N4 O2 S2 **Adduct(s):** H, Na, NH4, C3H5N2, radical

#	meas. m/z	theo. m/z	|Err|[ppm]	Sigma	Formula	Adduct	Adduct Mass
1	325.0781	325.0787	2.10	0.0063	C13H17N4O2S2	M+H	1.0078
1	347.0604	347.0607	0.80	0.0046	C13H16N4NaO2S2	M+Na	22.9898

Note: Sigma fits < 0.05 indicates high probability of correct MF

Figure S39. Mass Spectrum of **3l**

Mass Spectrometry
Analytical Services
School of Chemistry

The University of Nottingham

Sample-ID	h_meh_hm386	Lab	C13
Submitter	Hasnain Mehmood	Supervisor	Simon Woodward
Analysis Name	h_meh_hm386_613473_70_01_31277.d	Acquisition Date	4/12/2021 4:13:15 PM
Ionisation Mode	ESI Positive	Instrument	Bruker MicroTOF

+MS, 0.7-0.9min #42-53

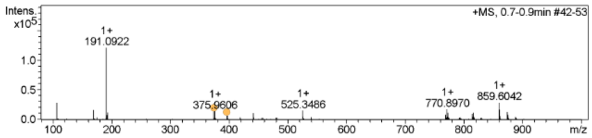

#	m/z	I %
1	107.0396	23.6
2	169.1100	13.0
3	191.0922	100.0
4	192.0942	6.1
5	193.0882	9.4
6	373.9627	10.9
7	375.9606	11.4
8	395.9441	4.8
9	397.9422	5.1
10	441.2973	8.6
11	525.3486	12.4
12	768.8985	6.4
13	770.8970	14.3
14	772.8943	9.3
15	814.2515	7.7
16	816.2501	9.1
17	859.6042	23.9
18	860.6073	13.6
19	873.6191	10.6
20	874.6231	6.3

Generate Molecular Formula Parameters

Charge	Tolerance	sigma limit	H/C Ratio	Electron Conf.	Nitrogen Rule	Chrom.BackGround	Calibration
+1	6 ppm	0.08	3 - 0	both	false	false	TRUE

Expected Formula C12 H12 Br1 N3 O2 S2 **Adduct(s):** H, Na, NH4, C3H5N2, radical

#	meas. m/z	theo. m/z	|Err|[ppm]	Sigma	Formula	Adduct	Adduct Mass
1	373.9627	373.9627	0.10	0.0128	C12H13BrN3O2S2	M+H	1.0078
1	395.9441	395.9447	1.40	0.0084	C12H12BrN3NaO2S2	M+Na	22.9898

Note: Sigma fits < 0.05 indicates high probability of correct MF

Figure S40. Mass Spectrum of **3m**

IR Spectrum of compounds 3a-m

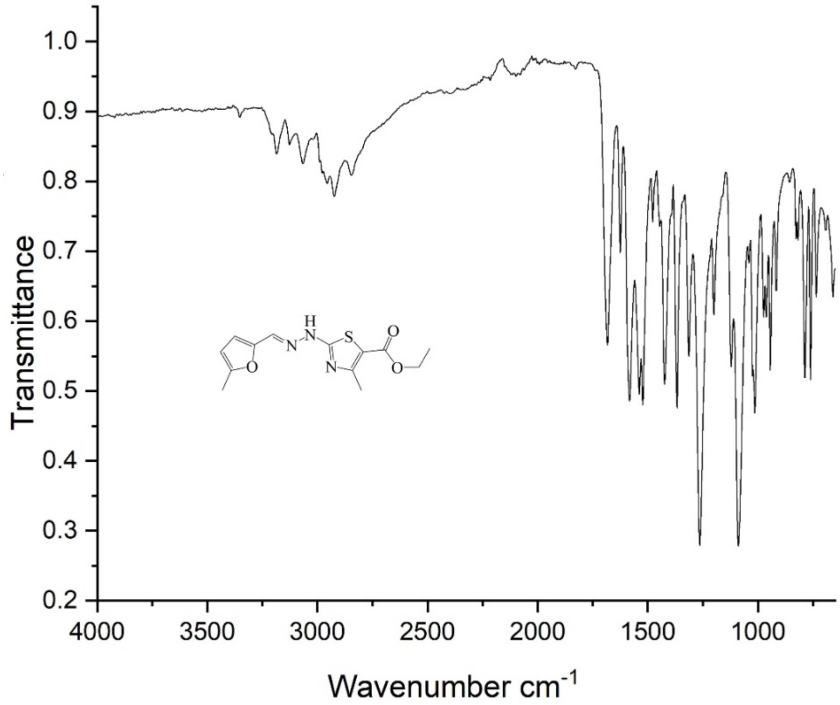

Figure S41. IR Spectrum of 3**a**

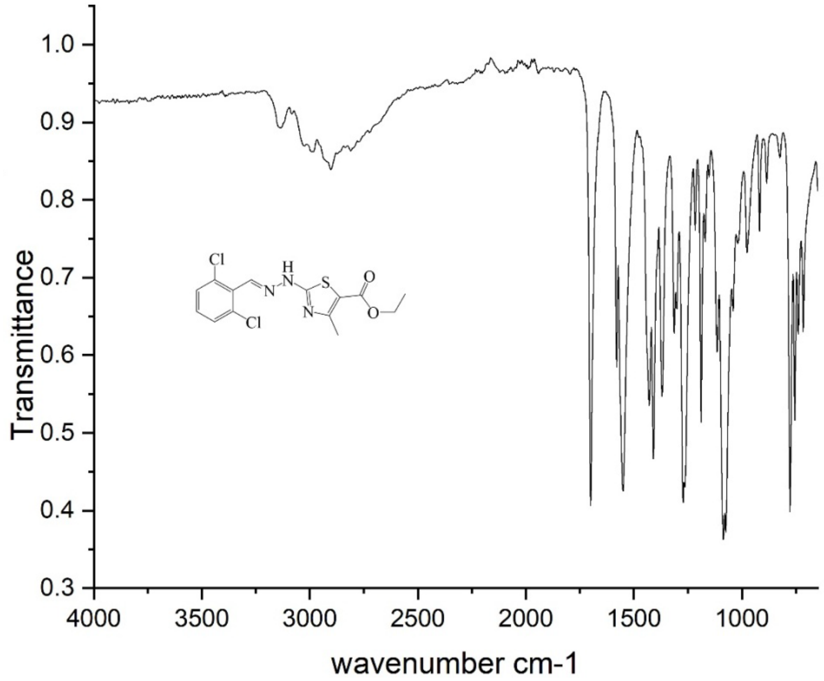

Figure S42. IR Spectrum of **3b**

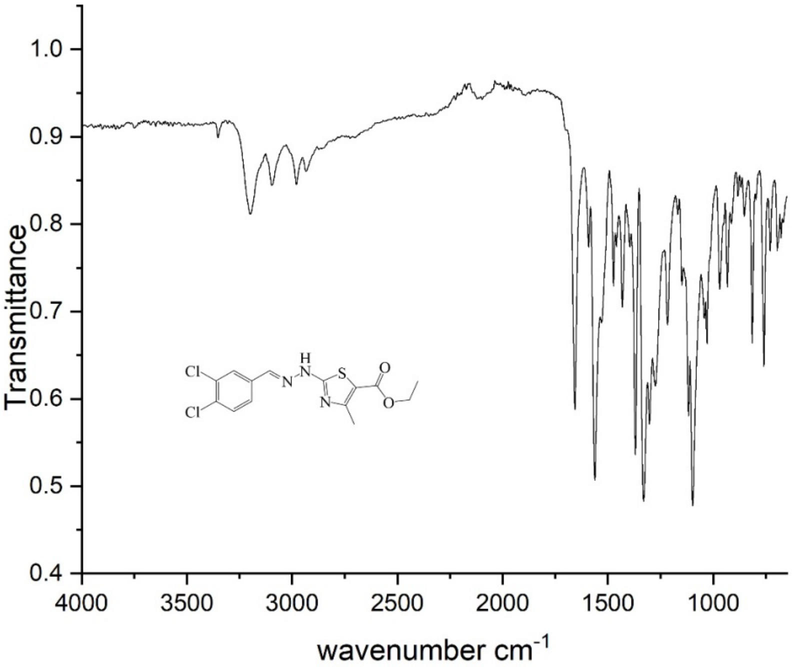

Figure S43. IR Spectrum of **3c**

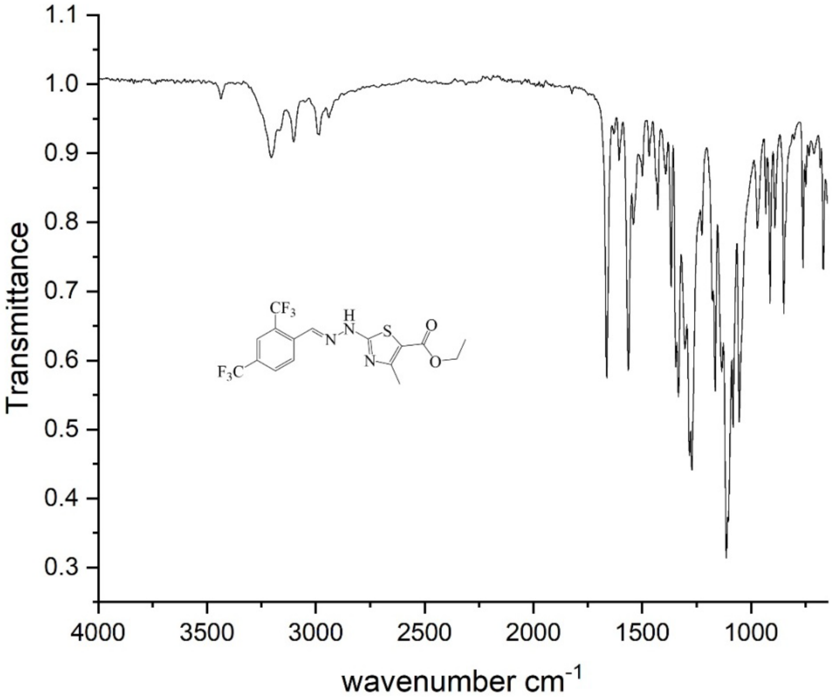

Figure S44. IR Spectrum of **3d**

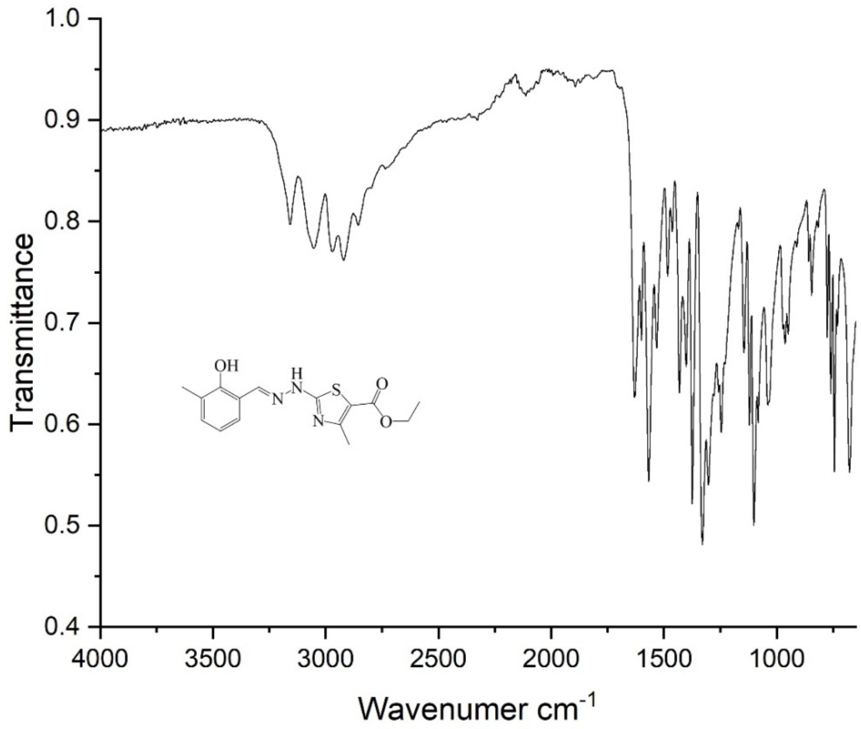

Figure S45. IR Spectrum of **3e**

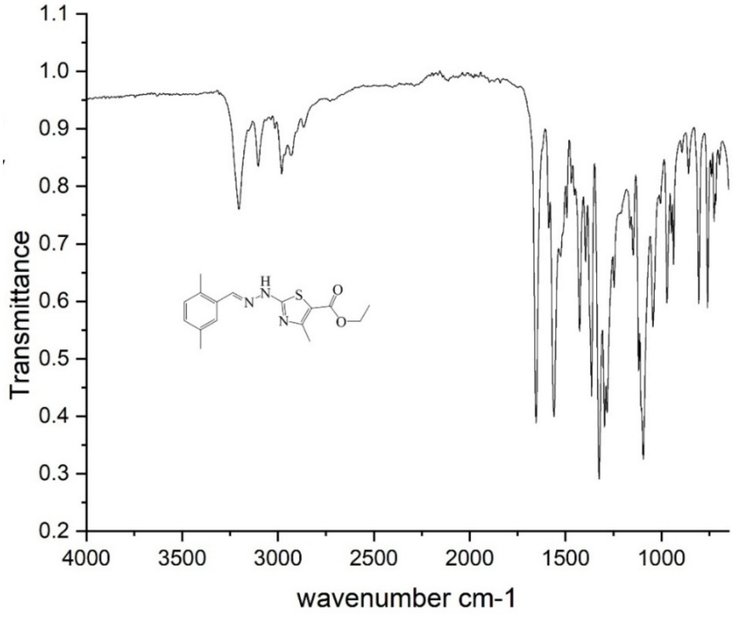

Figure S46. IR Spectrum of **3f**

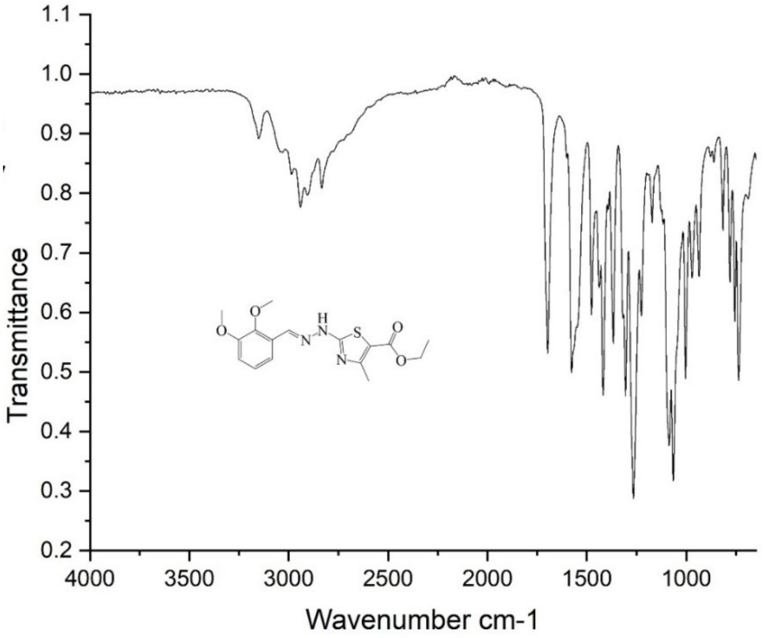

Figure S47. IR Spectrum of **3g**

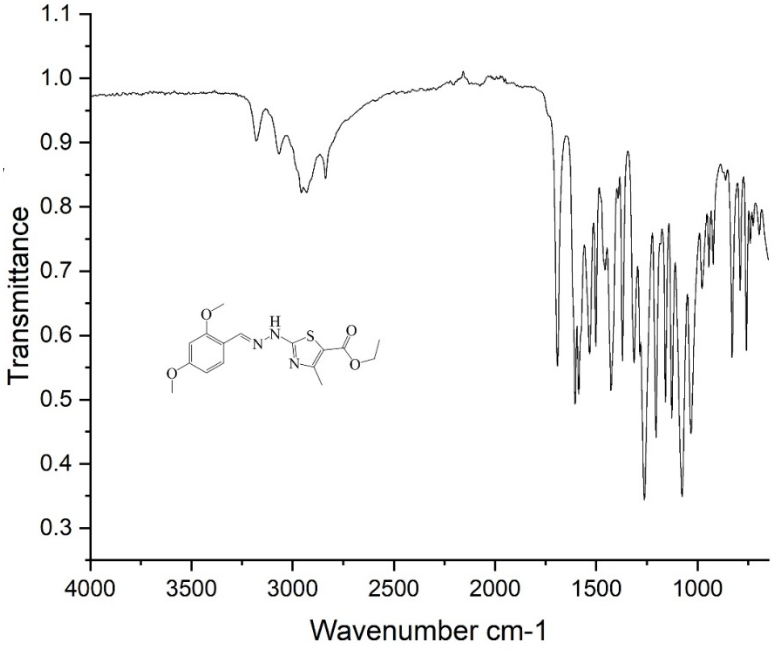

Figure S48. IR Spectrum of **3h**

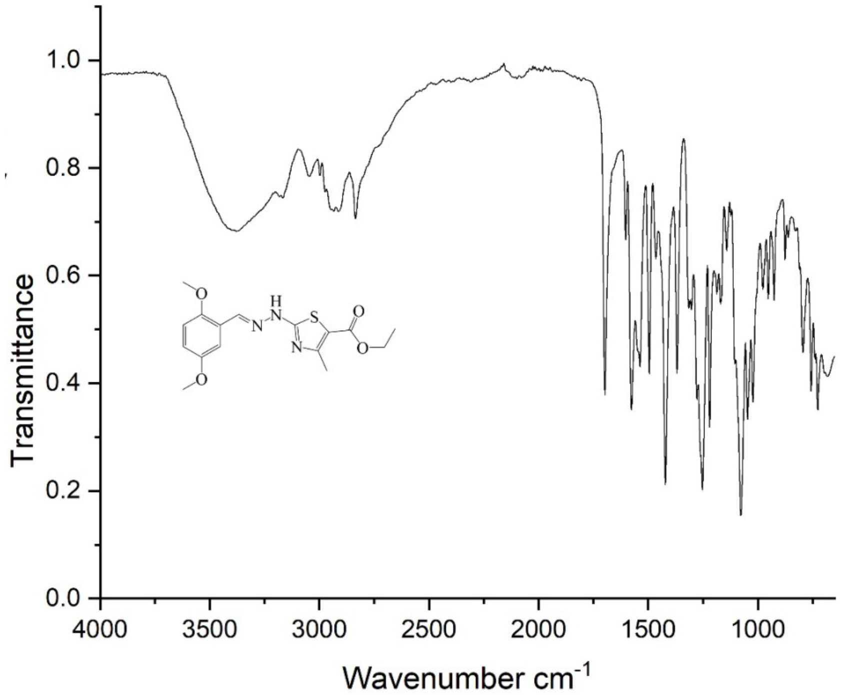

Figure S49. IR Spectrum of **3i**

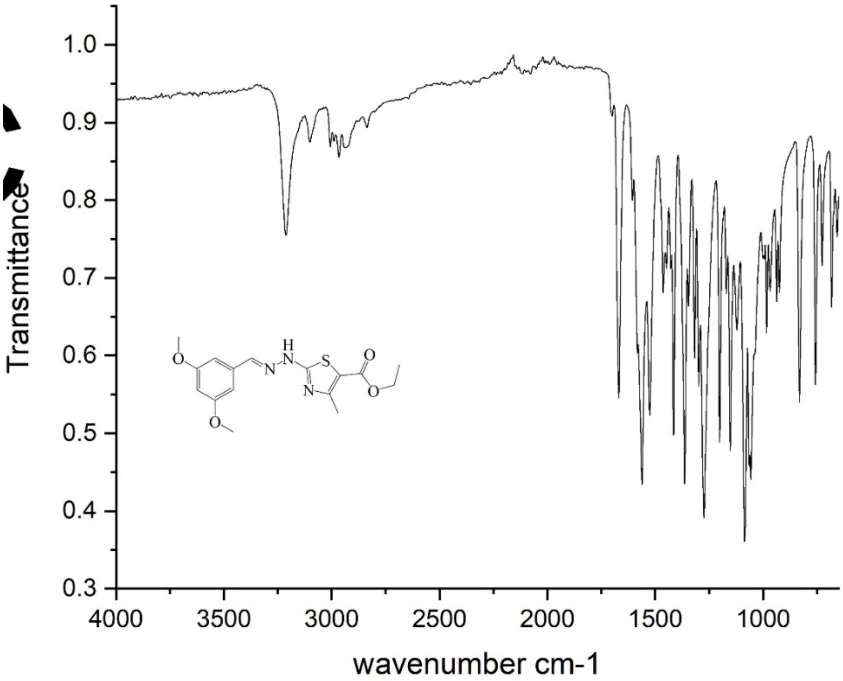

Figure S50. IR Spectrum of **3j**

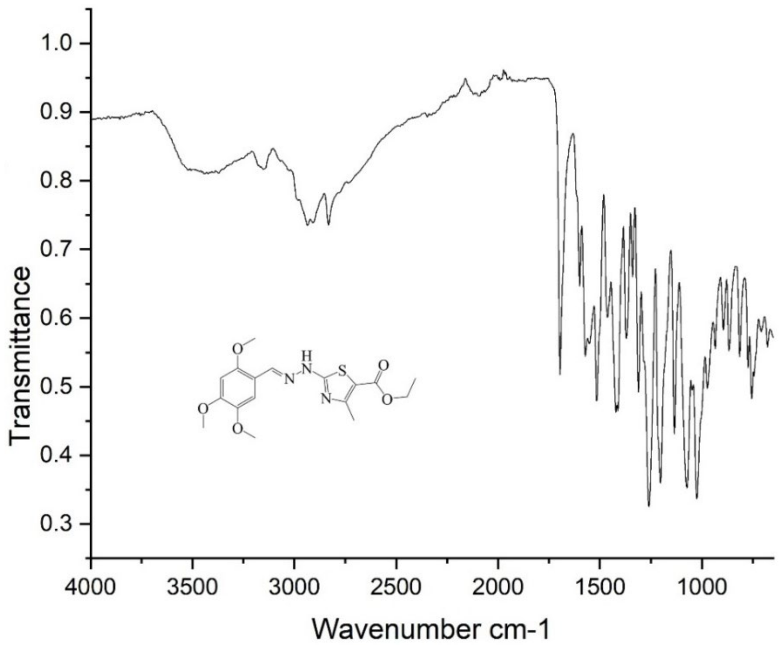

Figure S51. IR Spectrum of **3k**

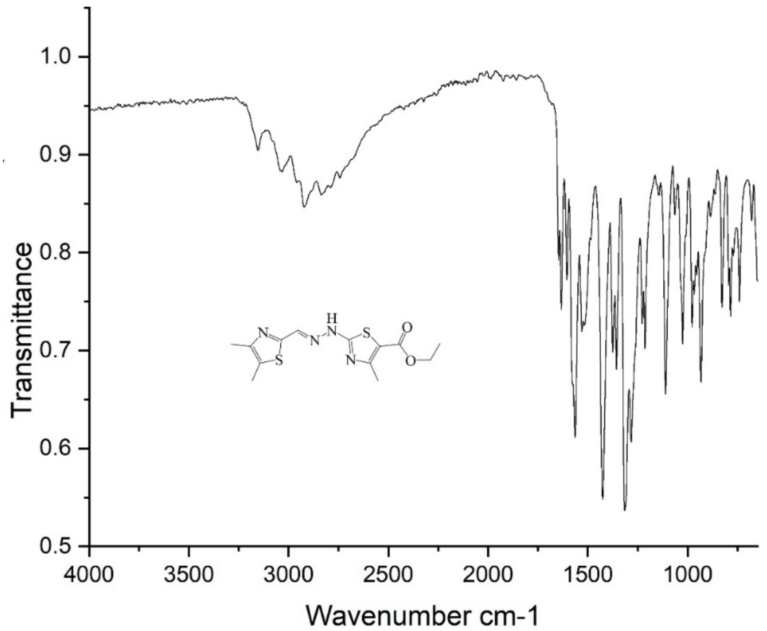

Figure S52. IR Spectrum of **3l**

Figure S53. 3D binding model of compounds **3b**, **3c**, **3h**, **3i**, **3k, 3m**.

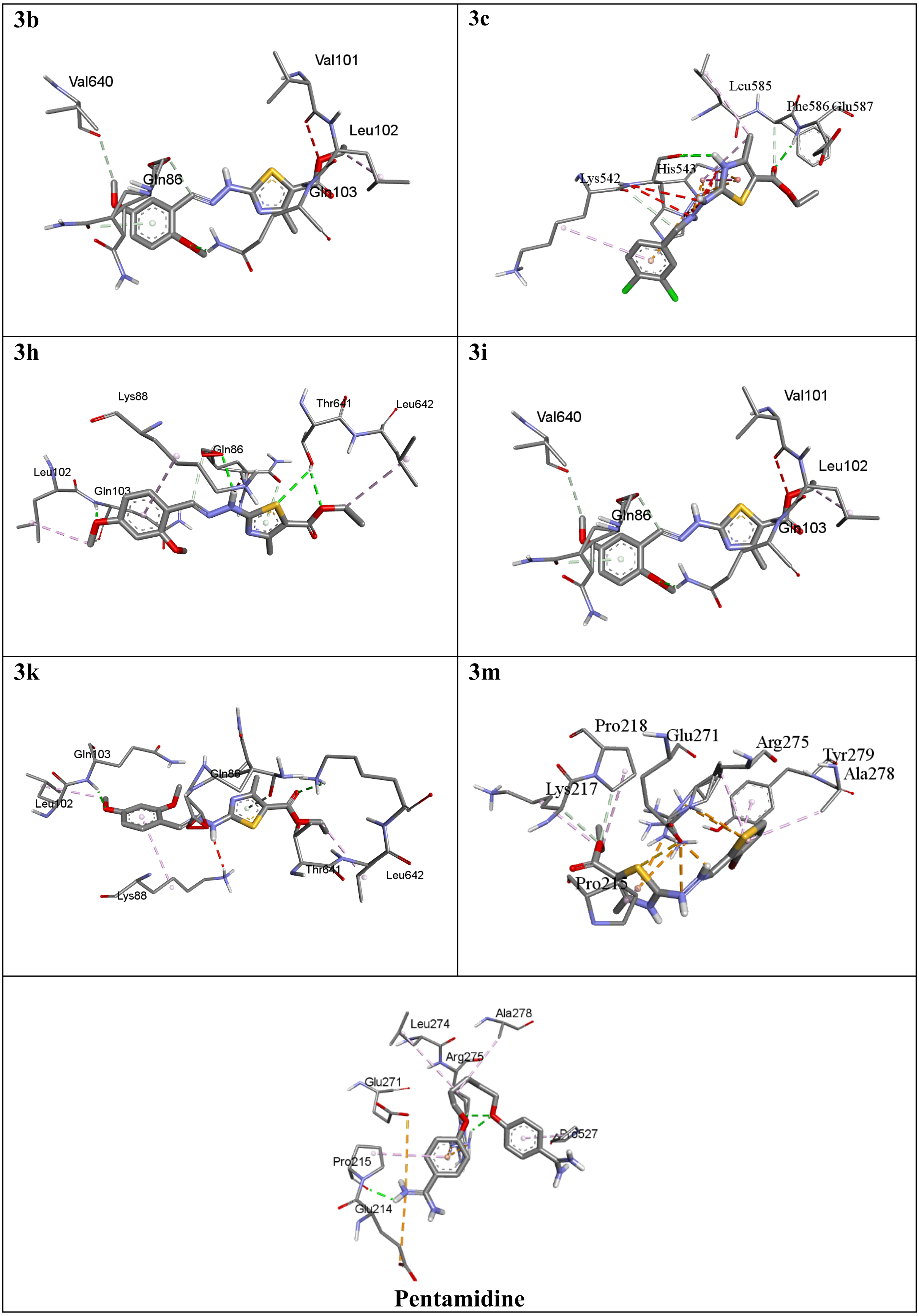

Figure S54. 2 D binding model of compounds **3b**, **3c**, **3h**, **3i**, **3k, 3m**.

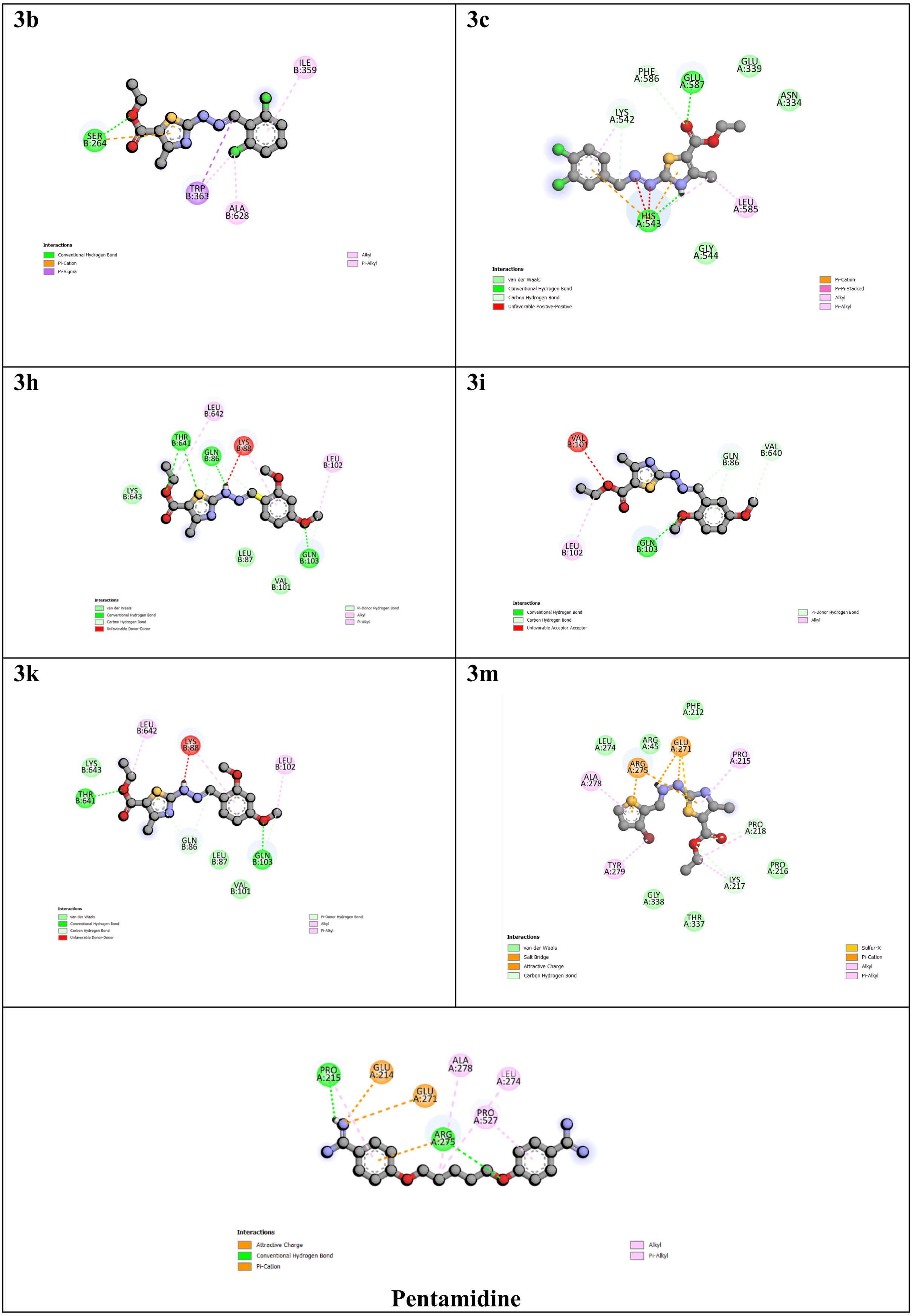

Figure S55: Cytotoxicity against NIH/3T3 cells for compounds **3a-m**

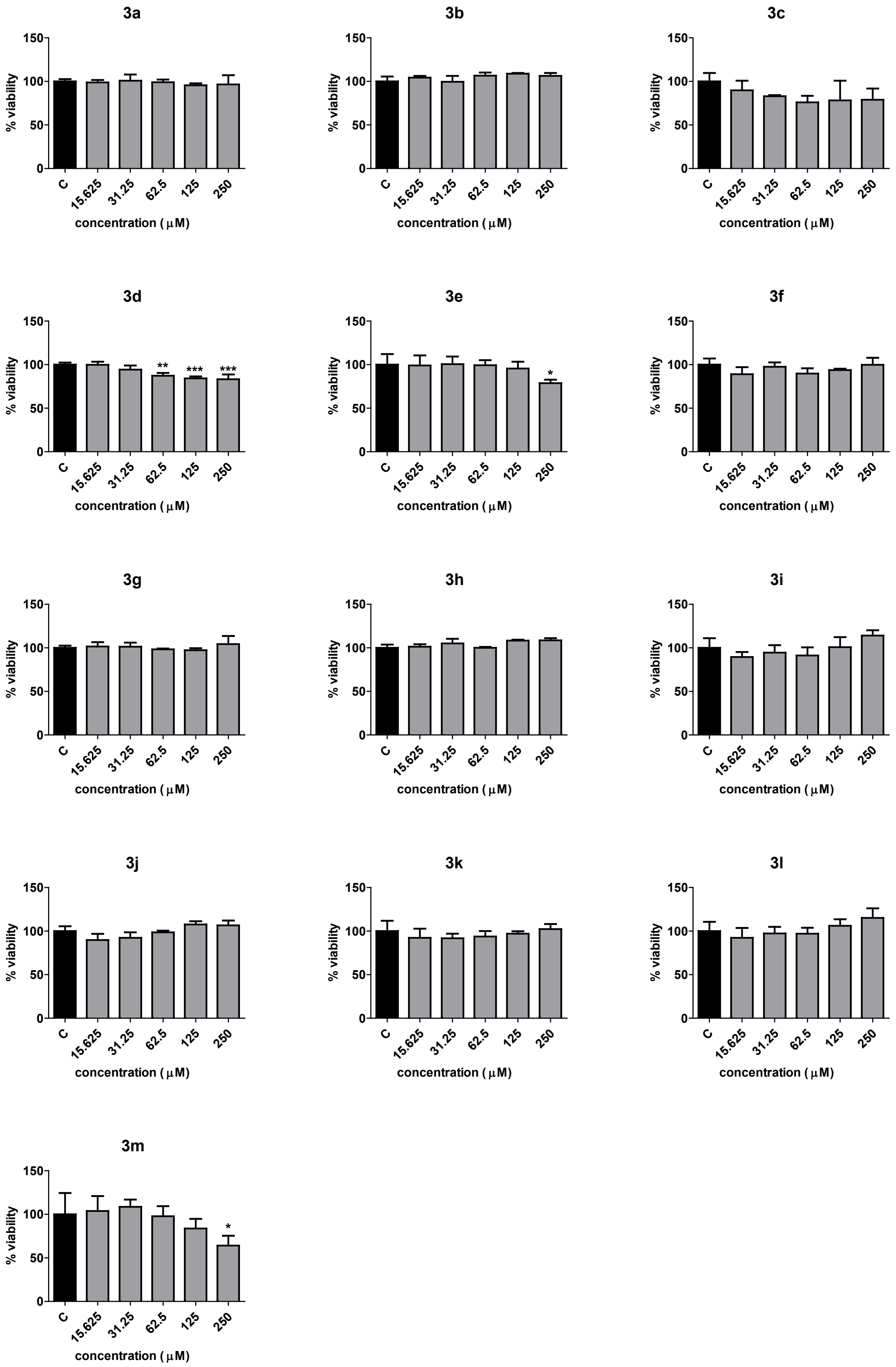

Figure S56: Antipromastigote activity for compounds **3a-m**

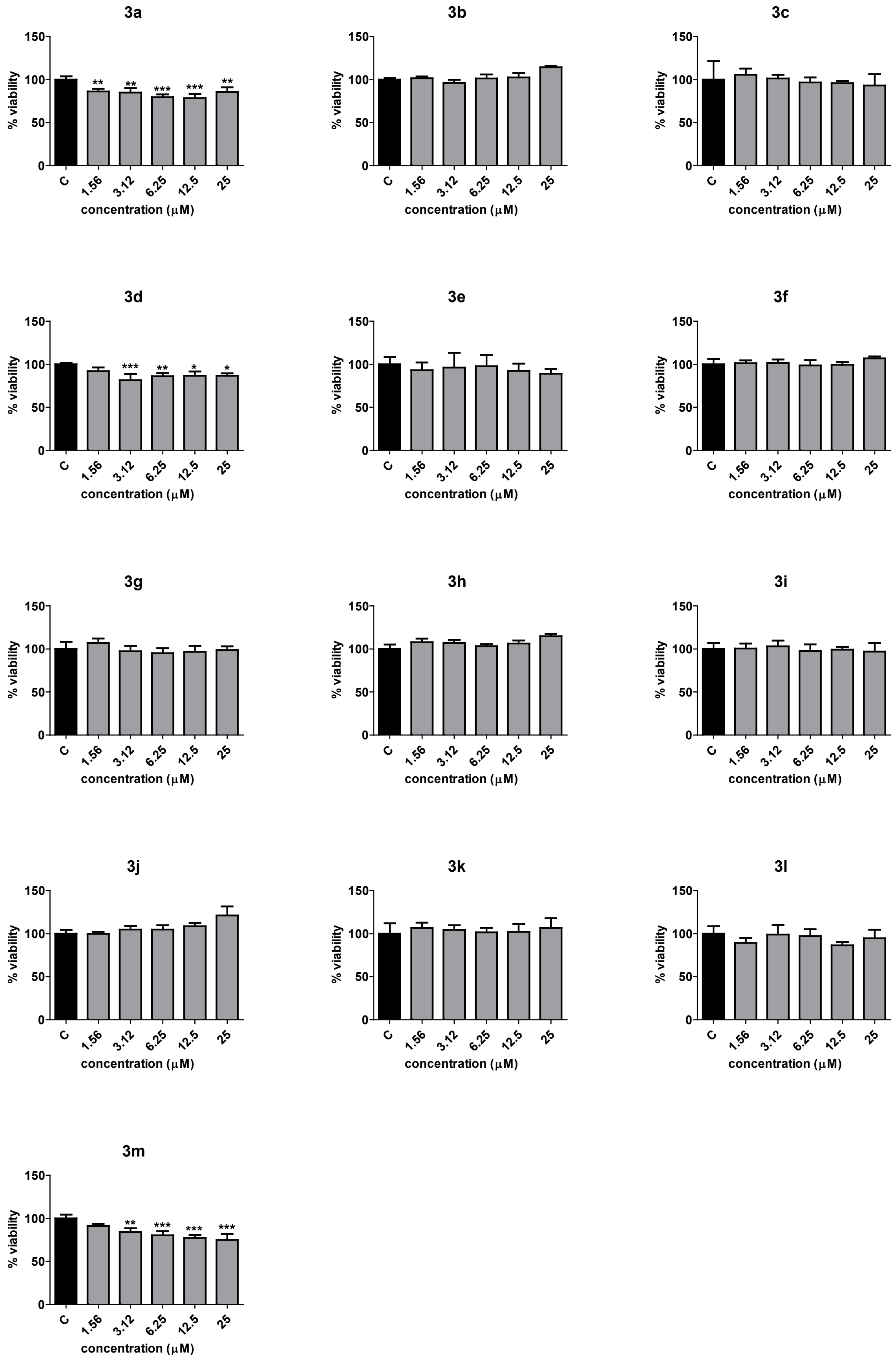

Figure S57: Antiamastigote activity for compounds **3a-m**

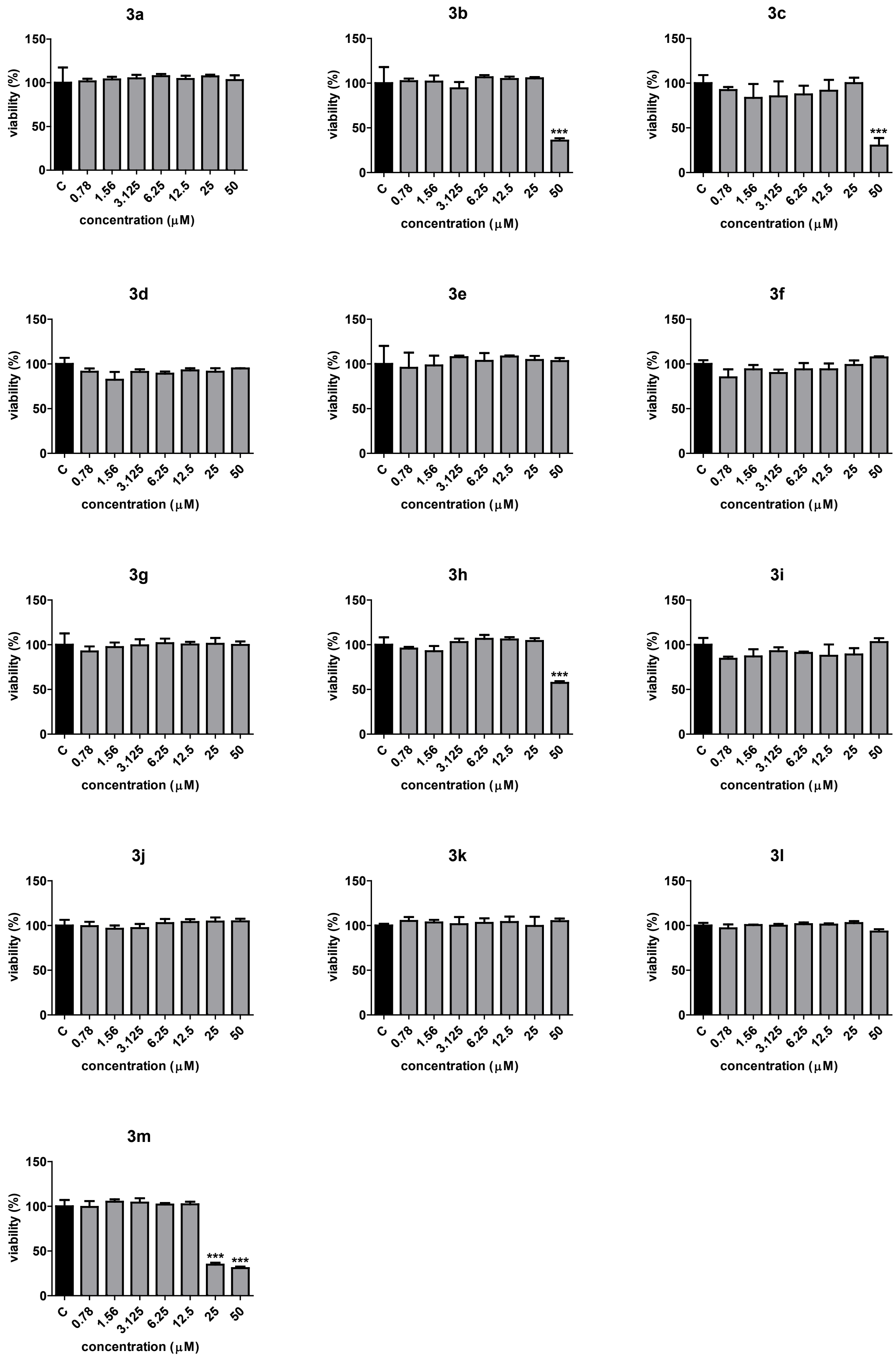

Figure S58: Cytotoxicity in different cells for reference drugs **amphotericin B** and **pentamidine**

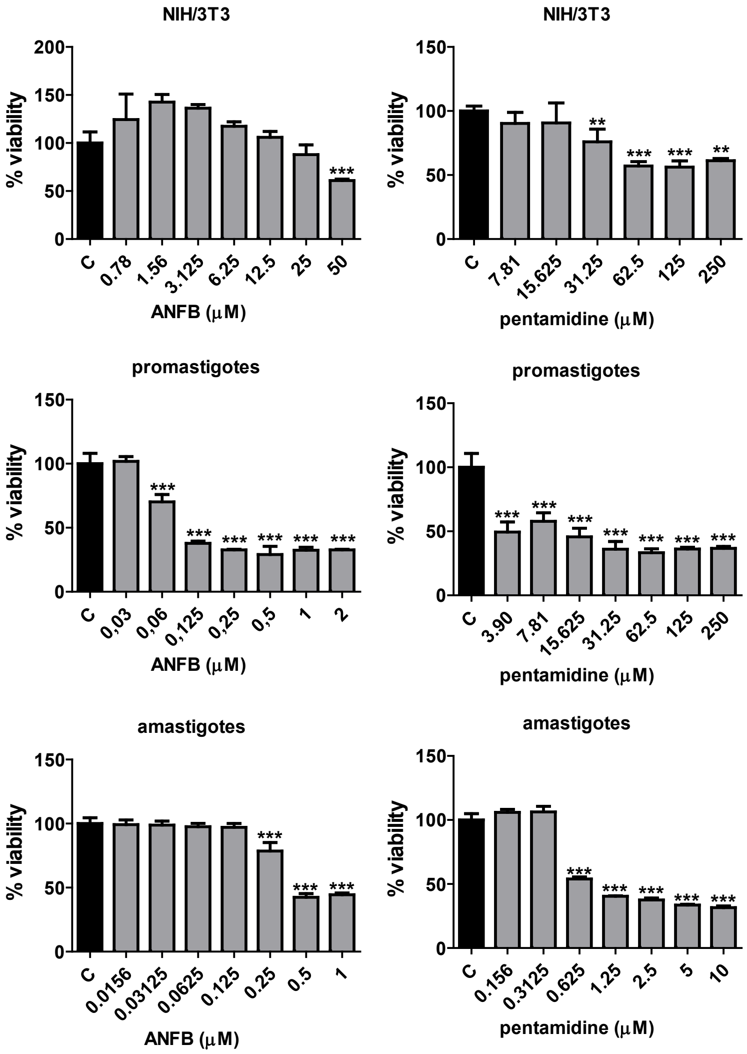

Supplement: Supplementary file 1 [file molecules-31-02278-s001.zip › molecules-4357716-supplementary.pdf]
